# Cyclic AMP Regulates Bacterial Persistence through Repression of the Oxidative Stress Response and SOS-Dependent DNA Repair in Uropathogenic *Escherichia coli*

**DOI:** 10.1128/mBio.02144-17

**Published:** 2018-01-09

**Authors:** Roberto C. Molina-Quiroz, Cecilia Silva-Valenzuela, Jennifer Brewster, Eduardo Castro-Nallar, Stuart B. Levy, Andrew Camilli

**Affiliations:** a Department of Molecular Biology and Microbiology, Tufts University School of Medicine, Boston, Massachusetts, USA; b Howard Hughes Medical Institute, Boston, Massachusetts, USA; c Center for Adaptation Genetics and Drug Resistance, Santiago, Chile; d Center for Bioinformatics and Integrative Biology, Facultad de Ciencias Biológicas, Universidad Andrés Bello, Santiago, Chile; Harvard Medical School

**Keywords:** CRP, DNA damage, SOS response, Tn-Seq, antibiotics, cAMP, dormancy, oxidative stress, persister cells

## Abstract

Bacterial persistence is a transient, nonheritable physiological state that provides tolerance to bactericidal antibiotics. The stringent response, toxin-antitoxin modules, and stochastic processes, among other mechanisms, play roles in this phenomenon. How persistence is regulated is relatively ill defined. Here we show that cyclic AMP, a global regulator of carbon catabolism and other core processes, is a negative regulator of bacterial persistence in uropathogenic *Escherichia coli*, as measured by survival after exposure to a β-lactam antibiotic. This phenotype is regulated by a set of genes leading to an oxidative stress response and SOS-dependent DNA repair. Thus, persister cells tolerant to cell wall-acting antibiotics must cope with oxidative stress and DNA damage and these processes are regulated by cyclic AMP in uropathogenic *E. coli*.

## IMPORTANCE

Bacterial persister cells are important in relapsing infections in patients treated with antibiotics and also in the emergence of antibiotic resistance. Our results show that in uropathogenic *E. coli*, the second messenger cyclic AMP negatively regulates persister cell formation, since in its absence much more persister cells form that are tolerant to β-lactams antibiotics. We reveal the mechanism to be decreased levels of reactive oxygen species, specifically hydroxyl radicals, and SOS-dependent DNA repair. Our findings suggest that the oxidative stress response and DNA repair are relevant pathways to target in the design of persister-specific antibiotic compounds.

## OBSERVATION

Urinary tract infections are a worldwide health concern. They are caused mainly by uropathogenic *Escherichia coli* (UPEC), which in most cases leads to chronic infection (1). It has been proposed that a persister subpopulation is responsible for generating relapsing infections (2). A full understanding of the molecular mechanisms and genetic regulation involved in the generation of persister cells is currently lacking.

The cyclic AMP (cAMP) receptor protein (CRP) was originally described as a global regulator of genes involved primarily in carbon catabolite repression (3). The DNA binding affinity of the CRP homodimer is increased upon cAMP binding. cAMP-CRP activates some genes while repressing others and is predicted to regulate 378 distinct promoters in *E. coli* K-12 (4). These include, as mentioned, genes involved in carbon catabolism but also genes involved in additional processes such as virulence, biofilm formation, and the SOS response (3, 5–7). cAMP-CRP has previously been implicated in the negative regulation of persistence in UPEC, wherein transposon insertions that disrupted *cyaA* (which encodes the cAMP synthase adenylate cyclase) and the promoter for *crp* resulted in increased survival upon exposure to the β-lactam antibiotic ampicillin (8). Consistent with this, another study found that reduction of the cAMP level via its hydrolysis by the cAMP-specific phosphodiesterase CpdA resulted in increased persistence (9). However, a more comprehensive understanding of the gene networks regulated by cAMP-CRP that contribute to persistence is lacking.

There are at least two classes of bacterial persisters, i.e., type I persisters, which are characterized by a dormant state generated, for example, during stationary phase and preexist at the time of an antibiotic challenge (10), and type II persisters, which are generated during exponential growth upon an antibiotic challenge and are believed to result from a combination of a dormant metabolic state and antibiotic challenge-specific mechanisms (10–12). In the present study, we explored persister formation in exponentially growing UPEC cultures exposed to different antibiotics, as well as the role of cAMP in this process.

**FIG 1:**
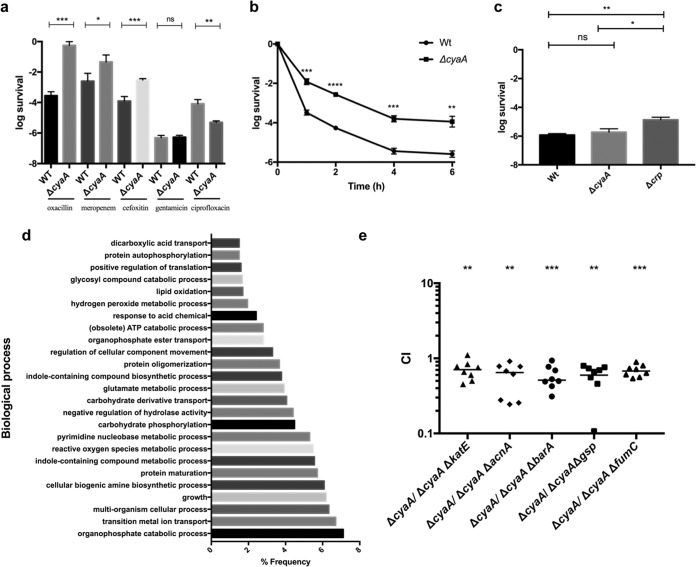
cAMP is an antibiotic-specific regulator of bacterial persistence, and this phenomenon depends on the oxidative stress response. (a) The ability to generate persister cells in UPEC cultures exposed to different antibiotics was assessed by CFU counting (*n* = 4). (b) Killing kinetics of *E. coli* cultures exposed to ampicillin and periodically assayed for viable cells (*n* = 4). (c) Bacterial survival upon exposure to ampicillin in the presence of exogenous cAMP (*n* = 4). (d) Functional classification of Tn-Seq data by GO analysis. A transposon library constructed in the Δ*cyaA* mutant background was exposed to ampicillin (*n* = 4), and the genes under negative selection were classified by biological process. (e) Assays of competition between the Δ*cyaA* single mutant and double mutants defective in *cyaA* and another gene detected by Tn-Seq screening. Overnight cultures of the single and double mutants were mixed in a 10:1 ratio, grown to exponential phase, and treated with ampicillin for 6 h. Aliquots were washed, and viable cells were plated and counted in LB medium with or without kanamycin (*n* = 4). Error bars denote standard errors. *, *P* = 0.05; **, *P* = 0.01; ***, *P* = 0.001; ****, *P* = 0.0001; ns, not significant.

Increased survival was observed in Δ*cyaA* mutant cultures exposed to the cell wall-acting antibiotics meropenem, cefoxitin, and oxacillin (Fig. 1a). We observed no effect after gentamicin exposure and the opposite effect, i.e., decreased survival, in ciprofloxacin-treated cultures. This suggests that the mechanisms affected by cAMP that are involved in the generation of persister cells are specific for different antibiotics and likely antibiotic target dependent, as shown previously (11, 12).

To further characterize the role that cAMP has in the generation of persister cells, we first compared the killing dynamics of the Δ*cyaA* mutant with those of the wild type (WT) in cultures exposed to ampicillin. After exposure to a lethal concentration of the antibiotic, we observed a classic biphasic killing curve, which is consistent with the generation of persister cells (13), and increased survival of the Δ*cyaA* mutant (Fig. 1b). Addition of exogenous cAMP restored WT killing kinetics to the Δ*cyaA* mutant (Fig. 1c), showing that the effect of the mutation is due to loss of cAMP production. The growth rate of the Δ*cyaA* mutant was lower than that of the WT strain (see Table S1 in the supplemental material). However, this by itself cannot explain the large increase in persister formation, since ampicillin kills growing bacteria, while metabolically dormant persister cells are spared. To further investigate the mechanism of cAMP inhibition on persister formation, we deleted *crp*, which encodes the cAMP receptor protein. Similar to what was observed with the Δ*cyaA* mutant, exponentially growing cultures of the Δ*crp* mutant exhibited 10-fold higher survival upon exposure to ampicillin than the WT, as described before (8). However, addition of exogenous cAMP did not restore WT killing kinetics to the Δ*crp* mutant (Fig. 1c), as would be expected if cAMP were acting through its receptor, CRP. These data are consistent with the hypothesis that cAMP-CRP mediates a major inhibitory effect on the formation of persisters in UPEC.

10.1128/mBio.02144-17.5 TABLE S1  Growth rate determination. Overnight cultures were diluted 1:1,000 in fresh medium, and 200-µl aliquots were placed in 96-well plates. Growth curves were generated by reading the plates every 5 min for 24 h with incubation at 37°C and constant shaking in a Synergy H1 microplate reader (BioTek). Growth determination was conducted with GrowthRates 2.1 software (B. G. Hall, H. Acar, A. Nandipati, and M. Barlow, Mol Biol Evol **31**:232–238, 2014, https://doi.org/10.1093/molbev/mst187). Download TABLE S1, DOCX file, 0.1 MB. Copyright © 2018 Molina-Quiroz et al. 2018 Molina-Quiroz et al. This content is distributed under the terms of the Creative Commons Attribution 4.0 International license.

To investigate how the Δ*cyaA* mutant generates a higher level of persister cells, we used transposon sequencing (Tn-Seq) of ampicillin-treated Δ*cyaA* mutant and WT cultures to identify genes involved in the phenotype. We identified 346 mutants in the WT background and 234 mutants in the Δ*cyaA* mutant background that showed decreased survival upon exposure to ampicillin (Tables S2 and S3, respectively). Strikingly, only four genes were common to both data sets, i.e., c1489, c2003, c2602, and c3075, which encode a hypothetical protein, fumarase C, hypothetical protein YegO, and the flavohemoprotein HmpA, respectively. That the vast majority of hits were unique to each data set is consistent with the hypothesis that cAMP plays a major regulatory role in persister formation.

10.1128/mBio.02144-17.6 TABLE S2  Mutants under negative selection displaying decreased fitness when a library constructed in a Δ*cyaA* mutant background was exposed to ampicillin. Download TABLE S2, DOCX file, 0.1 MB. Copyright © 2018 Molina-Quiroz et al. 2018 Molina-Quiroz et al. This content is distributed under the terms of the Creative Commons Attribution 4.0 International license.

10.1128/mBio.02144-17.7 TABLE S3  Mutants under negative selection displaying decreased fitness when a library constructed in a WT background was exposed to ampicillin. Download TABLE S3, DOCX file, 0.1 MB. Copyright © 2018 Molina-Quiroz et al. 2018 Molina-Quiroz et al. This content is distributed under the terms of the Creative Commons Attribution 4.0 International license.

There is increasing evidence that part of the damage generated by bactericidal antibiotics is mediated by reactive oxygen species (ROS), particularly hydroxyl radicals (OH˙) (14–18). We used gene ontology (GO) analysis to categorize the 234 genes identified in the Δ*cyaA* mutant background and determined that several pathways known to contribute to the oxidative stress response appear to play a role in persister formation in the Δ*cyaA* mutant (Fig. 1d). This included pathways for indole and glutamate metabolism (19, 20). Indeed, these pathways have previously been linked with tolerance to antibiotics (9, 21, 22). In addition, we hit genes related to the biosynthesis of biogenic amines, which have been shown to protect against oxidative damage (23, 24). We also identified genes involved in the regulation of translation, protein maturation, and protein oligomerization. This may be in response to the previously reported decrease in the ATP level after exposure to oxidative stress, which in turn causes inactivation of the DnaK chaperone and a subsequent increase in protein aggregation (25).

None of the genes associated with oxidative stress that were identified in the Δ*cyaA* mutant screening were identified in the WT strain screening (Tables S2 and S3). Instead, pathways related mainly to cell envelope biogenesis were found to be important (Fig. S1). These results indicate that different mechanisms are involved in the generation of persister cells in the WT and the Δ*cyaA* mutant under the conditions tested. Altogether, these results suggest that the increased generation of persister cells in the Δ*cyaA* mutant depends on different genes involved in oxidative stress response and therefore that cAMP-CRP plays a negative regulatory role in persister cell formation in the WT strain.

10.1128/mBio.02144-17.1 FIG S1  Functional classification of mutants with decreased fitness in the WT background. Functional classification of Tn-Seq data was done by GO analysis. A transposon library constructed in the WT background was exposed to ampicillin (*n* = 4), and the genes under negative selection were classified by biological process. Download FIG S1, TIF file, 14.1 MB. Copyright © 2018 Molina-Quiroz et al. 2018 Molina-Quiroz et al. This content is distributed under the terms of the Creative Commons Attribution 4.0 International license.

To explore the potential for a direct role of cAMP-CRP in the transcriptional regulation of genes identified in our screening, we used the PRODORIC tool (26) and determined that 84 (36%) of the 234 genes identified in the Δ*cyaA* mutant background have CRP binding boxes in their promoter regions (Table S4). This is a much higher percentage than expected by chance (7.5%) (4). Therefore, the regulation of persister cell formation exerted by cAMP-CRP appears to be mediated both by direct transcriptional regulation of genes and by indirect effects, consistent with previous reports (27, 28).

10.1128/mBio.02144-17.8 TABLE S4  PRODORIC prediction of genes identified by Tn-Seq with a predicted CRP binding box in the promoter region. Download TABLE S4, DOCX file, 0.1 MB. Copyright © 2018 Molina-Quiroz et al. 2018 Molina-Quiroz et al. This content is distributed under the terms of the Creative Commons Attribution 4.0 International license.

To validate the hits from the Tn-Seq screening, we conducted competition assays between the Δ*cyaA* mutant parent strain and double mutants corresponding to Δ*cyaA* and individual genes hit in our screening. We chose to validate *katE*, *acnA*, *barA*, *gsp*, and *fumC*, each of which is related to the oxidative stress response (29–31). Each double mutant exhibited a competitive defect when challenged with ampicillin (Fig. 1e). In contrast, single mutations in *katE*, *acnA*, *barA*, *gsp*, and *fumC* in the WT background did not impact the generation of persister cells (Fig. S2), thus supporting the cAMP dependence of these hits. Indeed, three of the five genes (*acnA*, *fumC*, and *gsp*) have a CRP binding box in their promoters (Table S4). The mild competitive defects exhibited by the double mutants are likely due to partial redundancy of the different mechanisms involved in the generation of persister cells in cultures exposed to ampicillin, consistent with a previous report (13).

10.1128/mBio.02144-17.2 FIG S2  Survival of bacteria with single mutations in genes identified by Tn-Seq in a Δ*cyaA* mutant background. Overnight cultures of WT and single mutant strains were used to inoculate fresh LB and grown for 1.5 h at 37°C with vigorous shaking. These cultures were exposed to ampicillin for 6 h and washed, and survival was assessed by CFU counting. Download FIG S2, TIF file, 14.1 MB. Copyright © 2018 Molina-Quiroz et al. 2018 Molina-Quiroz et al. This content is distributed under the terms of the Creative Commons Attribution 4.0 International license.

**FIG 2:**
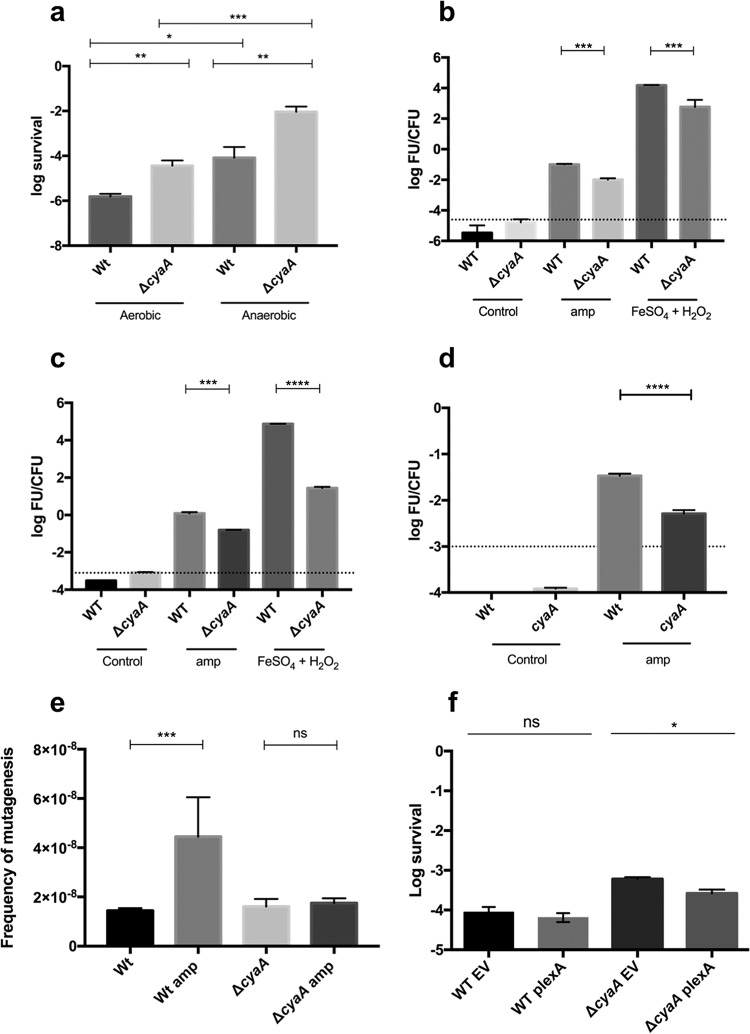
Decreased oxidative stress levels contribute to the increased survival in the Δ*cyaA* background by avoiding the generation of mutations in an SOS-dependent fashion. (a) The role of oxygen in the generation of persister cells was assessed by treating cultures under aerobic or anaerobic conditions. Cultures were grown to the exponential phase and challenged with ampicillin for 6 h aerobically or anaerobically (*n* = 4). Aliquots were washed and plated to assay viable counts. (b) Total ROS generation in ampicillin-treated cultures was assayed with the probe H_2_DCFDA and normalized by CFU counts after 1 h of treatment with ampicillin or with FeSO_4_ plus H_2_O_2_ (50 µM and 10 mM, respectively) as a positive control for OH˙ by the Fenton reaction (*n* = 3). (c) Generation of OH˙ in persister cells was assayed with the specific probe HPF and normalized by the number of survivors after 1 h of treatment with ampicillin or with FeSO_4_ plus H_2_O_2_ under aerobic conditions (*n* = 3) and normalized by CFU counts. (d) The contribution of oxygen to the generation of OH˙ in persister cells generated by the exposure of cultures to ampicillin under microaerophilic conditions was assayed with the specific probe HPF and normalized by the number of survivors after 1 h of treatment with ampicillin or with FeSO_4_ plus H_2_O_2_ in the presence of Oxyrase. (e) Frequency of mutagenesis was assayed in cells previously exposed to ampicillin for 1 h. Cultures were washed, grown overnight, and plated on LB agar plates supplemented with rifampin. (d) The contribution of the SOS response to the survival upon exposure to ampicillin was assessed by CFU counting with the WT and the Δ*cyaA* mutant transformed with a plasmid that generates an SOS^−^ phenotype because of a mutation in LexA (plexA) and compared to the empty vector (EV) (*n* = 4). Error bars denote standard errors. *, *P* = 0.05; **, *P* = 0.01; ***, *P* = 0.001; ****, *P* = 0.0001; ns, not significant.

To further evaluate the contribution of oxidative damage to the generation of persister cells, we challenged WT and Δ*cyaA* mutant cultures with ampicillin in the presence or absence of oxygen. There was ~100-fold greater ampicillin survival of both strains in the absence of oxygen than under aerobic conditions; however, the Δ*cyaA* mutant still exhibited approximately 100-fold greater survival than the WT strain (Fig. 2a). This result demonstrates the critical contribution of oxidative damage to the toxicity of ampicillin and the impact of ROS on the generation of persister cells.

We used the fluorescent probes 2',7'-dichlorodihydrofluorescein diacetate (H_2_DCFDA) and hydroxyphenyl fluorescein (HPF) to evaluate the role of cAMP in the generation of total ROS and OH˙, respectively, in cultures exposed to ampicillin. The total ROS and OH˙ levels were much higher in ampicillin-treated WT and Δ*cyaA* mutant cultures than in the respective untreated controls (Fig. 2b and c, respectively). However, there was ~10-fold less OH˙ generation in the Δ*cyaA* mutant than in the WT (Fig. 2c). Interestingly, the Δ*cyaA* deletion also results in a large decrease in the level of OH˙ generated through the Fenton reaction (32) in the presence of FeSO_4_ and H_2_O_2_ (Fig. 2c). These results suggest that oxidative stress responses are sufficiently induced in the Δ*cyaA* mutant to detoxify much of the ROS that is generated. This idea is supported by our Tn-Seq data showing that genes required to detoxify H_2_O_2_ are needed to cope with the oxidative damage generated by ampicillin exposure (Fig. 1d; Table S2). In addition, we observed a 5-fold increase in the survival of WT cultures exposed to ampicillin in the presence of dimethyl sulfoxide (DMSO), which is a known scavenger of OH˙ (33, 34). However, no difference was observed in that of the Δ*cyaA* mutant, suggesting that less OH˙ is produced in this strain during ampicillin exposure than in the WT (Fig. S3). No effect of DMSO itself on survival and/or the growth rate was observed.

10.1128/mBio.02144-17.3 FIG S3  A scavenger of OH˙ increases tolerance to ampicillin in WT UPEC. Overnight cultures of UPEC were used to inoculate LB. Survival was assessed by CFU counting of cultures exposed to ampicillin at 1,250 µg/ml for 6 h in the presence or absence of an OH˙ scavenger (5% DMSO) (*n* = 4). Cells were grown as mentioned in the text, and aliquots were washed and plated to determine survival. Download FIG S3, TIF file, 14.1 MB. Copyright © 2018 Molina-Quiroz et al. 2018 Molina-Quiroz et al. This content is distributed under the terms of the Creative Commons Attribution 4.0 International license.

We further evaluated the contribution of oxygen to the generation of OH˙ mediated by ampicillin by using Oxyrase, which scavenges oxygen to generate a microaerobic environment. We observed an ~10-fold higher level of OH˙ in the WT strain exposed to the antibiotic than in the Δ*cyaA* mutant (Fig. 2d). Similarly, we observed greater survival of Oxyrase-exposed cultures of both strains (Fig. S4) than of those treated with ampicillin under fully aerobic conditions, consistent with a contribution to cellular damage by ROS during ampicillin exposure. However, the Δ*cyaA* mutant still exhibited greater survival than the WT strain under microaerobic conditions (Fig. S4). These results further support our findings indicating a direct correlation between the lack of cAMP and an increased ability to cope with oxidative damage. Together, these results are in agreement with the induction of the oxidative stress response that is generated by the derepression of *rpoS* generated in cultures lacking *cyaA crp*, as previously reported (35).

10.1128/mBio.02144-17.4 FIG S4  Bacterial survival in cultures exposed to ampicillin under aerobic or microaerophilic conditions. Overnight cultures of UPEC were inoculated into LB. After 1.5 h of growing aerobically at 37°C, the cultures were treated with ampicillin or with FeSO_4_ plus H_2_O_2_ (50 µM and 10 mM) for 1 h in the presence or absence of Oxyrase. Survival was assessed by CFU counting (*n* = 4). Download FIG S4, TIF file, 14.1 MB. Copyright © 2018 Molina-Quiroz et al. 2018 Molina-Quiroz et al. This content is distributed under the terms of the Creative Commons Attribution 4.0 International license.

Since the main target of damage by OH˙ is DNA (32, 34, 36), we evaluated changes in the frequency of generation of rifampin-resistant mutants as an indicator of DNA damage. A higher mutagenesis rate was observed in the WT strain exposed to ampicillin than in the untreated control, as demonstrated previously (37–39). However, Δ*cyaA* mutant cultures challenged with ampicillin showed no detectable difference from the untreated control in the frequency of mutation (Fig. 2d), which is consistent with the reduced level of OH˙ in this strain (Fig. 2c).

Finally, it has been previously established that DNA damage generated by oxidative stress induces the SOS response (40, 41) and that this pathway is regulated by cAMP (42). We evaluated the contribution of the SOS response to survival upon exposure to ampicillin by using a plasmid containing an uncleavable version of the repressor LexA because of a mutation (K156R) that generates an SOS-deficient (SOS^−^) phenotype (43). We observed 4-fold less survival of a Δ*cyaA* mutant expressing the SOS^−^ phenotype than of a strain with the same background but a functional SOS response. No changes were observed in the WT strain under the same conditions (Fig. 2f). This result indicates that survival of the Δ*cyaA* mutant upon exposure to ampicillin requires an active SOS response and also suggests that the lower frequency of mutagenesis observed in cultures exposed to ampicillin (Fig. 2e) is dependent on SOS-dependent DNA repair.

Altogether, these results show that in cultures exposed to ampicillin and most likely to other cell wall-acting antibiotics, cAMP is an important negative regulator of persistence in UPEC. Our results also highlight the multifactorial and redundant nature of molecular mechanisms involved in the generation and survival of persister cells. We show that the phenotype of increased persister formation when cAMP is absent is mediated by the establishment of an active response leading to (i) decreased accumulation of ROS and (ii) coping with oxidative damage to DNA generated by hydroxyl radicals, which is dependent on an active SOS response. Thus, UPEC must deal not just with the damage generated by the antibiotic to its canonic target but also with the oxidative damage that bactericidal antibiotics generate as part of their lethality (14). In addition, our results provide mechanistic support for and new insight into the model in which *E. coli* can form persister cells by decreasing the concentration of indole in a cAMP-dependent fashion (9). The resulting persister state would allow UPEC to better tolerate beta-lactam antibiotic-mediated damage, to deal with the toxic effect of antibiotic-generated OH˙, and to decrease the occurrence of damaging mutations. Finally, these results suggest that a decreased mutagenesis rate might slow down the evolution of antibiotic-resistant strains associated with the persister subpopulation, as recently shown (44).

### Bacterial strains and growth conditions.

Cultures of UPEC CFT073 were grown with vigorous shaking at 37°C in LB broth Miller (referred to here as LB). Similarly, LB was supplemented with oxacillin at 2,500 µg/ml, meropenem at 0.3 µg/ml, cefoxitin at 320 µg/ml, ciprofloxacin at 0.13 µg/ml, gentamicin at 30 µg/ml, kanamycin at 50 µg/ml, or at ampicillin at 1,250 µg/ml when required. To generate microaerophilic conditions, we grew *E. coli* cultures for 1 h in the presence of 100 µl of Oxyrase/ml of broth medium.

Mutant strains were constructed by recombination of PCR products with the pKM208 plasmid containing the lambda Red recombineering system (45). Recombinant clones were selected in kanamycin at 30 µg/ml and then restreaked onto LB agar plates containing kanamycin at 50 µg/ml. The presence of each mutation was confirmed by PCR amplification and then transferred to the WT genetic background with phage φEB49 (46). The strains and plasmids used in this study and the sequences of the primers used in this study are listed in Tables S5 and S6, respectively.

10.1128/mBio.02144-17.9 TABLE S5  Strains used in this study. Download TABLE S5, DOCX file, 0.1 MB. Copyright © 2018 Molina-Quiroz et al. 2018 Molina-Quiroz et al. This content is distributed under the terms of the Creative Commons Attribution 4.0 International license.

10.1128/mBio.02144-17.10 TABLE S6  Primers used in this study. Download TABLE S6, DOCX file, 0.1 MB. Copyright © 2018 Molina-Quiroz et al. 2018 Molina-Quiroz et al. This content is distributed under the terms of the Creative Commons Attribution 4.0 International license.

### Persister assays.

Overnight cultures were diluted 100-fold in fresh LB and incubated at 37°C with shaking for 1.5 h to the mid-exponential growth phase (typically reaching ~3 × 10^8^ CFU/ml). The bacteria were then exposed to ampicillin at 1,250 µg/ml (100 times the MIC for the WT and 50 times the MIC for the Δ*cyaA* mutant) for 6 h at 37°C with aeration. CFU counts were determined by plating on LB agar supplemented with 20 mM MgSO_4_ and sodium pyruvate at 2 mg/ml (persister plates) to improve plating efficiency as described previously (47). Survival was determined by dividing the number of CFU/ml of the culture after 6 h of exposure to antibiotics by the number of CFU/ml before antibiotic addition and converted logarithmically.

For persister assays performed under anaerobiosis, overnight cultures were grown under anaerobic conditions and then used to inoculate fresh morpholinepropanesulfonic acid (MOPS)-buffered LB (100 mM MOPS, pH 7.4) supplemented with 20 mM xylose to avoid catabolite repression, as shown previously (48).

### Construction of transposon library.

We used a derivative of the pDL1098 mTn*10 in vivo* transposition vector (49) called pDL1093, in which the spectinomycin resistance gene was replaced with a kanamycin resistance gene, to generate transposon insertion libraries in UPEC. We constructed an ~17,000-transposon insertion library in the *cyaA*::FRT background as previously described (49). Briefly, pDL1093 was moved into the Δ*cyaA* mutant by mating. An overnight culture of one single colony was then grown in LB supplemented with chloramphenicol at 10 μg/ml and kanamycin at 50 μg/ml at 30°C, which is a temperature permissive for both plasmid replication and repression of the Tn*10* transposase gene. Next, a flask prewarmed to 40°C and containing fresh LB supplemented with kanamycin at 50 μg/ml was inoculated with 100 µl of the overnight culture and incubated for 24 h at 40°C, which both derepresses transposase expression and is nonpermissive for plasmid replication. Aliquots were stored at −80°C in 20% glycerol.

### Tn-Seq.

Frozen stocks of the library were thawed and used to inoculate 30 ml of fresh LB in a 1:100 dilution and grown overnight. The next day, 1 ml of culture was used to inoculate 100 ml of fresh LB and grown for 1.5 h to generate the exponential growth phase input library.

To select for persisters, the input library was incubated for an additional 6 h at 37°C in the presence of ampicillin at 1,250 µg/ml for the library constructed in the Δ*cyaA* mutant background and at 125 µg/ml for the WT strain library because of the difference between the MICs for the two strains (12.5 µg/ml for the WT and 25 µg/ml for the Δ*cyaA* mutant). The survivors were washed with sterile phosphate-buffered saline (PBS) and grown in LB overnight to generate the output library. Genomic DNA was extracted from the input and output libraries for sequencing of transposon junctions by the homopolymer tail-mediated ligation PCR method (50). Samples were sequenced in an Illumina HiSeq 2500, and the relative abundance of each transposon insertion in the input and output samples was determined with the Galaxy platform available at Tufts University. To identify mutants with fitness changes, the Dval genome value was used. Dval genome value is calculated as follows: (number of reads of gene X/total number of reads)/(size of gene X)/(size of the genome). Mutants were considered to be under negative selection if (i) each gene had at least three unique insertions in all input samples, (ii) the Dval genome value in all of the input samples was ≥0.01, and (iii) a median survival index (Dval genome output − input) of ≤0.2 was observed.

### Bioinformatic analysis.

Gene and locus IDs from Tn-Seq results were used to obtain predicted protein sequences for GO annotation (*E. coli* NCBI reference no NC_004431). Briefly, protein sequences were compared against the Bacteria database from the eggNOG project by using Hidden Markov Models (version 4.5.1) (51). The resulting GO terms were summarized as in REVIGO by reducing the redundancy of terms (small 0.5; UniProt; SimRel) (52).

The presence of CRP binding boxes in the promoter regions of the genes identified by Tn-Seq was determined with the regulon analysis tool of PRODORIC (26).

### Competition assays.

Overnight cultures of the single mutant (*cyaA*::FRT) and double mutants (*cyaA*::FRT/other gene from Tn-Seq screening; Kan^r^) were mixed in a 10:1 ratio, and the mixture was used to inoculate fresh LB at a 1:100 dilution and grown for 1.5 h at 37°C to the exponential growth phase. Cultures were exposed to ampicillin at 1,250 µg/ml for 6 h at 37°C with vigorous shaking. One-milliliter aliquots were washed once with sterile PBS, serially diluted, and plated on persister plates and persister plates supplemented with 50 µg/ml kanamycin (LB-kan) to assay the survival of mutants. To determine the number of cells of the Δ*cyaA* mutant, we calculated the difference between the total number of cells (CFU counts in LB plates) and the number of double mutant cells (CFU counts in LB-kan plates). No difference in the efficiency of plating of the double mutants was observed between LB-kan and LB plates. The competitive index (CI) was calculated as follows: [CFU of double mutant/CFU of single mutant (output)]/[CFU of double mutant/CFU of single mutant (input)]. Values were converted logarithmically, and the statistical significance of differences was determined with a two-tailed Student *t* test comparing CI values with an ideal value of 1.

### Fluorimetric detection of total ROS and hydroxyl radicals.

UPEC cultures were grown as described above and challenged for 1 h with ampicillin at 1,250 µg/ml or with 50 µM FeSO_4_ and 10 mM H_2_O_2_ to generate hydroxyl radicals by the Fenton reaction (positive control) as previously described (32). Total ROS and hydroxyl radical levels were assessed with H_2_DCFDA and HPF, respectively, as previously described (14, 53). Fluorescence of cultures was determined in a Synergy H1 microplate reader (BioTek) fluorimeter. Values were normalized to CFU counts obtained by serially diluting and plating washed aliquots as mentioned earlier.

### Determination of changes in the frequency of mutagenesis.

Overnight cultures were grown and challenged for 1 h with ampicillin at 1,250 µg/ml. Cultures were washed three times with fresh LB and grown for ~1,000 generations in 200 ml of LB by overnight growth to enrich them for rifampin-resistant mutants. The next day, 250 µl of each culture was plated on plates of LB agar and LB agar supplemented with rifampin at 100 µg/ml as previously reported (54). The frequency of mutagenesis was calculated by dividing the total number of rifampin-resistant cells by the total number of cells.

### Determination of survival of SOS^−^ bacteria.

A plasmid containing an uncleavable version of LexA (43) was cloned and transformed into the WT and the Δ*cyaA* mutant. Overnight cultures of both strains were used to inoculate fresh LB-kan (30 µg/ml) supplemented with 100 µM isopropyl-β-d-thiogalactopyranoside (IPTG) and grown for 1.5 h as described above. Next, each culture was exposed to ampicillin for 6 h, washed once with sterile PBS, and plated on LB plates (supplemented with MgSO_4_ and sodium pyruvate as stated above) without kanamycin. Survival was determined by CFU counting.

### Statistical analysis.

Statistical significance was determined with a two-tailed Student *t* test.
