# Supplementary material for: Cyclic AMP Regulates Bacterial Persistence through Repression of the Oxidative Stress Response and SOS-Dependent DNA Repair in Uropathogenic Escherichia coli
Source: mBio. 2018 Jan 9;9(1):e02144-17. doi: 10.1128/mBio.02144-17 (PMC5760743; doi:10.1128/mBio.02144-17)
Supplement: TABLE S1 [file mbo001183668st1.docx]

**Table S1**

| Locus | Protein | Gene | Median SI |
| --- | --- | --- | --- |
|  |  |  |  |
| c0023 | Hypothetical protein |  | 0.000846756 |
| c0041 | Carbamoyl-phosphate synthase large chain | *carB* | 0.028154568 |
| c0050 | FixA protein | *fixA* | 0.017410186 |
| c0070 | RNA polymerase associated protein | *hepA* | 0.063353636 |
| c0093 | Probable activator protein in leuABCD operon | *leuO* | 0.003497887 |
| c0139 | Putative Transposase for IS629 |  | 0.009058834 |
| c0170 | Outer membrane usher protein htrE precursor | *htrE* | 0.000646382 |
| c0178 | DnaK suppressor protein | *dksA* | 0.008865458 |
| c0259 | Hypothetical protein |  | 0.007805841 |
| c0266 |  |  | 0.02848917 |
| c0286 | Conserved hypothetical protein |  | 0.081251741 |
| c0345 | Putative member of ShlA/HecA/FhaA exoprotein family |  | 0.008765325 |
| c0350 | Pic serine protease precursor |  | 0.006900945 |
| c0361 | Putative cytoplasmic membrane export protein |  | 0.022797584 |
| c0363 | Putative RTX family exoprotein A gene |  | 0.002272228 |
| c0393 | Haemoglobin protease |  | 0.001572558 |
| c0415 | Putative adhesin | *eaeH* | 0.001536727 |
| c0440 | Hypothetical protein yahE | *yahE* | 0.001356733 |
| c0469 | Hypothetical protein yaiP |  | 0.009264015 |
| c0622 | Glyoxylate carboligase | *gcl* | 0.018469407 |
| c0624 | 2-hydroxy-3-oxopropionate reductase | *ybbQ* | 0.039504226 |
| c0630 | Allantoate amidohydrolase | *ylbB* | 0.001514445 |
| c0673 | Enterobactin synthetase component F | *entF* | 0.010779875 |
| c0688 | Hypothetical aminotransferase ybdL | *ybdL* | 0.177600187 |
| c0690 | Hypothetical protein ybdN |  | 0.003445767 |
| c0712 | Anaerobic C4-dicarboxylate transporter dcuC | *dcuC* | 0.004241571 |
| c0741 | Hypothetical protein |  | 0.091459824 |
| c0757 | Hypothetical protein |  | 0.081809768 |
| c0760 | Hypothetical protein ygdQ |  | 0.002236103 |
| c0846 | Hypothetical protein ybhH | *ybhH* | 0.002366803 |
| c0847 | Hypothetical protein ybhI | *ybhI* | 0.013356342 |
| c0893 | Hypothetical protein ybiO | *ybiO* | 0.005475457 |
| c0918 | Hypothetical protein yliE | *yliE* | 0.001644726 |
| c0924 | Penicillin-binding protein 6 precursor | *dacC* | 0.013338733 |
| c1052 | Hypothetical protein ycaI | *ycaI* | 0.018053593 |
| c1081 | Dihydroorotate dehydrogenase | *pyrD* | 0.005999551 |
| c1127 | Putative electron transport protein yccM | *yccM* | 0.008748225 |
| c1160 | Hypothetical protein ycdP | *ycdP* | 0.003537859 |
| c1162 | Hypothetical lipoprotein ycdR precursor | *ycdR* | 0.000814786 |
| c1163 | Hypothetical protein ycdS precursor | *ycdS* | 0.000239884 |
| c1170 | Hypothetical protein yfjI |  | 0.02174905 |
| c1232 | Probable microcin H47 secretion ATP-binding protein | *mchF* | 0.003346093 |
| c1242 | F1C fimbrial usher | *focD* | 0.000572131 |
| c1247 | Putative Regulatory protein | *focX* | 0.027687596 |
| c1251 | IroE protein | *iroE* | 0.00415947 |
| c1253 | ATP binding cassette (ABC) transporter homolog | *iroC* | 0.00781548 |
| c1267 | Hypothetical protein |  | 0.003261097 |
| c1448 | Putative capsid assembly protein of prophage |  | 0.00352246 |
| c1468 | Hypothetical protein |  | 0.009323253 |
| c1489 | Hypothetical protein |  | 0.003468279 |
| c1519 | Prophage lambda integrase |  | 0.003211035 |
| c1541 | Putative single-stranded DNA binding protein of prophage |  | 0.008701086 |
| c1569 | Putative DNA packaging protein of prophage; terminase large subunit |  | 0.006946462 |
| c1590 | Putative tail component of prophage |  | 0.017376164 |
| c1591 | Hypothetical protein |  | 0.004104844 |
| c1610 | Conserved hypothetical protein |  | 0.01151215 |
| c1632 | UmuC protein | *umuC* | 0.012138448 |
| c1692 | Hypothetical protein |  | 0.024283033 |
| c1698 | Hypothetical protein ychK | *ychK* | 0.039747701 |
| c1726 | Tryptophan synthase beta chain | *trpB* | 0.004751306 |
| c1730 | Anthranilate synthase component I | *trpE* | 0.006796662 |
| c1745 | Aconitate hydratase 1 | *acnA* | 0.000909936 |
| c1758 | Hypothetical protein yciW | *yciW* | 0.067307438 |
| c1764 | Acriflavine resistance protein B |  | 0.001073208 |
| c1780 | Putative sucrose phosphorylase | *ycjM* | 0.00578661 |
| c1791 | Outer membrane protein G precursor | *ompG* | 0.002749177 |
| c1800 | Conserved hypothetical protein |  | 0.003444401 |
| c1802 | Hypothetical transcriptional regulator ycjZ |  | 0.001896095 |
| c1810 | Hypothetical protein |  | 0.012808983 |
| c1815 | Hypothetical protein ydaM | *ydaM* | 0.00375688 |
| c1821 | Unknown protein 2D_000B3L from 2D-page | *ynaF* | 0.019831858 |
| c1823 | Probable pyruvate-flavodoxin oxidoreductase | *ydbK* | 0.013124207 |
| c1831 |  | *ydbA_2* | 0.00246737 |
| c1840 | ATP-dependent helicase hrpA | *hrpA* | 0.010775825 |
| c1842 | Aldehyde dehydrogenase A | *aldA* | 0.001484881 |
| c1880 | Putative conserved protein |  | 0.014118975 |
| c1886 | Hypothetical protein |  | 0.000998453 |
| c1888 | Conserved hypothetical protein |  | 0.029030499 |
| c1901 | Nitrite extrusion protein 2 | *narU* | 0.001368073 |
| c1921 | Amino acid antiporter | *xasA* | 0.010314822 |
| c1922 | Glutamate decarboxylase beta | *gadB* | 0.000920128 |
| c1923 | Probable zinc protease pqqL | *pqqL* | 0.005348495 |
| c1924 | Hypothetical protein yddB | *yddB* | 0.005042662 |
| c1925 | Hypothetical ABC transporter ATP-binding protein yddA | *yddA* | 0.003763323 |
| c1926 | Hypothetical protein ydeM | *ydeM* | 0.005678924 |
| c1928 | Hypothetical transcriptional regulator ydeO | *ydeO* | 0.022322998 |
| c1930 | Hypothetical protein ydeP | *ydeP* | 0.0009275 |
| c1934 | Outer membrane usher protein fimD precursor |  | 0.005531744 |
| c1935 | Chaperone protein fimC precursor |  | 0.003455013 |
| c1958 | Putative conserved protein |  | 0.004188303 |
| c1962 | Hypothetical protein ydeE | *ydeF* | 0.001752439 |
| c1963 | Hypothetical protein ydeH | *ydeH* | 0.007475113 |
| c1964 | Peptidyl-dipeptidase dcp | *dcp* | 0.007838571 |
| c1969 | Hypothetical metabolite transport protein ydfJ | *ydfJ* | 0.005386534 |
| c1970 | Starvation sensing protein rspB | *rspB* | 0.001264328 |
| c1971 | Starvation sensing protein rspA | *rspA* | 0.034028021 |
| c1987 | Hypothetical transport protein ynfM | *ynfM* | 0.003195179 |
| c2003 | Fumarate hydratase class II | *fumC* | 0.009743327 |
| c2008 | Glucuronide carrier protein | *uidB* | 0.015366495 |
| c2009 | Beta-glucuronidase | *uidA* | 0.003413827 |
| c2021 | Electron transport complex protein rnfC |  | 0.01905031 |
| c2065 | Putative ferredoxin-like protein ydhX |  | 0.01013003 |
| c2067 | Hypothetical protein ydhV | *ydhV* | 0.005363012 |
| c2082 | Hypothetical protein ydiJ | *ydiJ* | 0.002298812 |
| c2085 | Hypothetical transport protein ydiM | *ydiM* | 0.006858677 |
| c2086 | Hypothetical transport protein ydiN | *ydiN* | 0.002058426 |
| c2089 | Hypothetical protein ydiF | *ydiF* | 0.006289603 |
| c2097 | Hypothetical protein ydiD | *ydiD* | 0.002095867 |
| c2098 | Phosphoenolpyruvate synthase | *ppsA* | 0.001219313 |
| c2131 | Catalase HPII | *katE* | 0.001739647 |
| c2134 | Cel operon repressor | *celD* | 0.002200658 |
| c2136 | PTS system, cellobiose-specific IIC component | *celB* | 0.002601882 |
| c2152 | Hypothetical protein ydjY precursor | *ydjY* | 0.013148845 |
| c2163 | Hypothetical protein |  | 0.004464748 |
| c2164 | Chaperone protein hscC | *ybeW* | 0.007662127 |
| c2173 | Hypothetical metabolite transport protein ydjE | *ydjE* | 0.000369842 |
| c2190 | Hypothetical protein yeaI | *yeaI* | 0.001658814 |
| c2243 | Hypothetical protein yebT | *yebT* | 0.00428303 |
| c2256 | Protease II | *ptrB* | 0.002394125 |
| c2348 | Outer membrane porin protein nmpC precursor |  | 0.018768775 |
| c2367 | Flagellar biosynthetic protein fliR | *fliR* | 0.022643705 |
| c2424 | Putative peptide synthetase |  | 0.007406639 |
| c2440 | Hypothetical protein |  | 0.003004102 |
| c2458 | Putative peptide synthetase |  | 0.029501895 |
| c2459 | Putative peptide synthetase |  | 0.003838062 |
| c2460 | Putative polyketide synthase |  | 0.003418748 |
| c2461 | Hypothetical protein |  | 0.005744902 |
| c2467 | Putative 3-hydroxyacyl-CoA dehydrogenase |  | 0.016521017 |
| c2470 | Putative peptide/polyketide synthase |  | 0.011350594 |
| c2485 | Hypothetical protein ybdN |  | 0.089479335 |
| c2518 | TonB dependent receptor |  | 0.001906946 |
| c2520 | Conserved hypothetical protein |  | 0.02534641 |
| c2555 | UDP-glucose 6-dehydrogenase | *ugd* | 0.005638343 |
| c2594 | Hypothetical protein yegE | *yegE* | 0.00111941 |
| c2602 | Hypothetical protein yegO | *yegO* | 0.007865061 |
| c2636 | Hypothetical outer membrane usher protein yehB precursor | *yehB* | 0.004616572 |
| c2643 | Putative conserved protein |  | 0.004827558 |
| c2644 | Hypothetical protein yehI | *yehI* | 0.011570296 |
| c2649 | Hypothetical protein yehL | *yehL* | 0.001978025 |
| c2651 | Hypothetical protein yehP | *yehP* | 0.13915984 |
| c2683 | Galactoside transport ATP-binding protein mglA | *mglA* | 0.002630872 |
| c2701 | Hypothetical sugar kinase yeiC | *yeiC* | 0.0008989 |
| c2770 | Putative membrane protein |  | 0.002477826 |
| c2775 | Hypothetical protein yfaL precursor | *yfaL* | 0.002567191 |
| c2813 | Hypothetical protein yfbK | *yfbK* | 0.002386557 |
| c2833 | Hypothetical protein yfbS | *yfbS* | 0.006787206 |
| c2895 | yapH homolog |  | 0.004821931 |
| c2899 | D-serine deaminase activator | *dsdC* | 0.003043757 |
| c2906 | Sensor protein evgS precursor | *evgS* | 0.009844969 |
| c2907 | Hypothetical protein yfdE | *yfdE* | 0.001220783 |
| c2933 | Hypothetical protein yfeA | *yfeA* | 0.026021817 |
| c2939 | Xanthosine permease | *xapB* | 0.001814902 |
| c2941 | Hypothetical protein yfeN | *yfeN* | 0.00570918 |
| c2995 | AegA protein | *yffG* | 0.003947624 |
| c3020 | Exopolyphosphatase | *ppx* | 0.008738828 |
| c3031 | SinH homolog |  | 0.02326453 |
| c3043 | Hypothetical lipoprotein yfhM precursor | *yfhM* | 0.001465725 |
| c3075 | Flavohemoprotein | *hmpA* | 0.007587825 |
| c3114 | ClpB protein | *clpB* | 0.01478738 |
| c3150 | Hypothetical protein |  | 0.001605494 |
| c3154 | Putative tail component of prophage |  | 0.001237686 |
| c3209 | Succinate-semialdehyde dehydrogenase (NADP+) | *gabD* | 0.001643073 |
| c3235 | Hypothetical protein ygaZ | *ygaZ* | 0.002401674 |
| c3320 | Alkaline phosphatase isozyme conversion protein precursor | *iap* | 0.011967474 |
| c3332 | Hypothetical oxidoreductase ygcW | *ygcW* | 0.001855787 |
| c3349 | Sensor protein barA | *barA* | 0.001985176 |
| c3397 | Hypothetical protein |  | 0.009788164 |
| c3415 | Protease III precursor | *ptr* | 0.001811695 |
| c3456 | Hypothetical protein ygfK | *ygfK* | 0.006914198 |
| c3465 | Putative purine permease ygfU | *ygfU* | 0.002458992 |
| c3514 | Putative oxidoreductase |  | 0.003511041 |
| c3515 | PTS system, mannitol (Cryptic)-specific IIBC component | *cmtA* | 0.061278868 |
| c3570 | Hemolysin A | *hlyA* | 0.010103237 |
| c3582 | PapX protein | *papX* | 0.030787514 |
| c3583 | PapG protein | *papG* | 0.00229294 |
| c3607 | Hypothetical protein |  | 0.033256692 |
| c3652 | Hypothetical protein yfjI |  | 0.045029998 |
| c3694 | Hypothetical protein |  | 0.01171054 |
| c3712 | Putative saframycin Mx1 synthetase B |  | 0.013100616 |
| c3725 | Glutathionylspermidine synthase | *gsp* | 0.022067389 |
| c3753 | Ureidoglycolate dehydrogenase |  | 0.014826998 |
| c3792 | Hypothetical outer membrane usher protein yqiG precursor | *yqiG* | 0.018581484 |
| c3810 | Hypothetical transcriptional regulator ygiP | *ygiP* | 0.002172505 |
| c3833 | Evolved beta-galactosidase alpha-subunit | *ebgA* | 0.005758092 |
| c3836 | Hypothetical transporter ygjI | *ygjI* | 0.040211417 |
| c3837 | Hypothetical protein ygjJ precursor | *ygjJ* | 0.001746439 |
| c3863 | Hypothetical transcriptional regulator yhaJ | *yhaJ* | 0.043883617 |
| c3876 | Tdc operon transcriptional activator | *tdcA* | 0.004409468 |
| c4095 | Probable general secretion pathway protein C | *yheE* | 0.0018603 |
| c4096 | Probable general secretion pathway protein D precursor | *yheF* | 0.00176447 |
| c4184 | Protein yhgF | *yhgF* | 0.005206398 |
| c4186 | Ferrous iron transport protein B | *feoB* | 0.027782151 |
| c4212 | Hypothetical outer membrane usher protein ycbS precursor | *ycbS* | 0.030986441 |
| c4215 | Glycogen phosphorylase | *glgP* | 0.002478627 |
| c4286 | Hypothetical ABC transporter ATP-binding protein yhiH | *yhiH* | 0.012714905 |
| c4289 | Hypothetical protein yhiM | *yhiM* | 0.001910333 |
| c4342 | Cellulose synthase operon protein C | *yhjL* | 0.098777398 |
| c4369 | Biotin sulfoxide reductase | *bisC* | 0.006298445 |
| c4382 | Hypothetical protein yiaA | *yiaA* | 0.004821226 |
| c4384 | Xylulose kinase | *xylB* | 0.002777917 |
| c4424 | Putative adhesin |  | 0.003125363 |
| c4495 | Hexuronate transporter |  | 0.004275459 |
| c4530 | Propionate kinase | *tdcD* | 0.021819324 |
| c4588 | Hypothetical protein yicO | *yicO* | 0.013049206 |
| c4602 | Putative symporter yidK | *yidK* | 0.001218755 |
| c4603 | Hypothetical transcriptional regulator yidL | *yidL* | 0.010983898 |
| c4631 | Tryptophanase | *tnaA* | 0.010751687 |
| c4642 | Putative outer membrane protein yieC precursor |  | 0.001224172 |
| c4758 | PTS system, glucose-specific IIBC component |  | 0.022883989 |
| c4759 | Transketolase 1 |  | 0.006633597 |
| c4822 | Hypothetical protein yihM | *yihM* | 0.000741423 |
| c4829 | Hypothetical protein |  | 0.001221965 |
| c4832 | Hypothetical protein yihX | *yihX* | 0.033721812 |
| c4849 | Putative glycoporin |  | 0.001081081 |
| c4850 | Hypothetical protein yiiL | *yiiL* | 0.00359704 |
| c4878 | Glycerol kinase | *glpK* | 0.002780196 |
| c4910 | Formate acetyltransferase 2 | *pflD* | 0.00349776 |
| c4920 | Starvation sensing protein rspA |  | 0.002828101 |
| c4976 | 5-methyltetrahydrofolate--homocysteine methyltransferase | *metH* | 0.00095095 |
| c5052 | Hypothetical protein yjcC | *yjcC* | 0.030101907 |
| c5093 | D-allose transport ATP-binding protein alsA | *yjcW* | 0.006167079 |
| c5114 | Hypothetical protein yjdA | *yjdA* | 0.001265535 |
| c5140 | Lysine decarboxylase, inducible | *cadA* | 0.00282429 |
| c5144 | Hypothetical protein |  | 0.040014053 |
| c5178 | Putative Transposase for IS629 |  | 0.027516183 |
| c5179 | PapG protein | *papG_2* | 0.007764484 |
| c5202 | Regulatory protein | *pgtC* | 0.1201262 |
| c5244 | Hypothetical transporter yjeM | *yjeM* | 0.030169641 |
| c5298 | Hexuronate transporter |  | 0.000667559 |
| c5341 | Mg(2+) transport ATPase, P-type 1 | *mgtA* | 0.003923236 |
| c5347 | Hypothetical protein |  | 0.02408162 |
| c5421 | Hypothetical protein |  | 0.001452048 |
| c5479 | Soluble lytic murein transglycosylase precursor | *slt* | 0.053238001 |
| c5621 |  | *ydbA_1* | 0.061170355 |
