# Supplementary material for: Cyclic AMP Regulates Bacterial Persistence through Repression of the Oxidative Stress Response and SOS-Dependent DNA Repair in Uropathogenic Escherichia coli
Source: mBio. 2018 Jan 9;9(1):e02144-17. doi: 10.1128/mBio.02144-17 (PMC5760743; doi:10.1128/mBio.02144-17)
Supplement: TABLE S2 [file mbo001183668st2.docx]

**Table S2**

| Locus | Protein | Gene | Median SI |
| --- | --- | --- | --- |
|  |  |  |  |
| c0035 |  | yaaF | 0.169330263 |
| c0051 |  |  | 0.195750094 |
| c0060 | Putative toxin of gyrase inhibiting toxin-antitoxin system |  | 0.024520735 |
| c0068 | DnaJ-like protein djlA | yabH | 0.136398271 |
| c0120 | Hypothetical protein |  | 0.047483628 |
| c0129 | AmpD protein | ampD | 0.020577527 |
| c0154 | Hypoxanthine phosphoribosyltransferase | hpt | 0.081316845 |
| c0173 | Hypothetical protein |  | 0.140353664 |
| c0176 | Poly(A) polymerase | pcnB | 0.023056578 |
| c0183 | Penicillin-binding protein 1B | mrcB | 0.000773797 |
| c0194 | Vitamin B12 transport protein btuF precursor | yadT | 0.040229081 |
| c0251 | Ribonuclease HI | rnhA | 0.008120996 |
| c0258 | Unknown in ISEc8 |  | 0.026862663 |
| c0272 | Hypothetical protein yeeT |  | 0.188497633 |
| c0304 | Hypothetical protein |  | 0.015005064 |
| c0309 | Conserved hypothetical protein |  | 0.195407438 |
| c0326 | Hypothetical protein |  | 0.18393109 |
| c0338 | Hypothetical protein |  | 0 |
| c0396 | Insertion element IS1 1/2/3/5/6 protein insA |  | 0.107792211 |
| c0400 | Hypothetical protein yagV precursor | yagV | 0.024247068 |
| c0406 | Putative 50S ribosomal protein L36 |  | 0.035689947 |
| c0464 | Hypothetical protein yaiM | yaiM | 0.031128453 |
| c0472 | Taurine-binding periplasmic protein precursor | tauA | 0.166076446 |
| c0473 | Taurine transport ATP-binding protein tauB | tauB | 0.042369671 |
| c0488 | Hypothetical protein |  | 0.011994602 |
| c0495 | Shikimate kinase II | aroL | 0.023743836 |
| c0497 | Hypothetical protein |  | 0 |
| c0502 | Recombination associated protein rdgC | rdgC | 0.174510048 |
| c0522 | Hypothetical lipoprotein yajI precursor | yajI | 0.076107839 |
| c0544 | AmpG protein | ampG | 0.169273623 |
| c0545 | Hypothetical protein |  | 0.077623102 |
| c0549 | Hypothetical protein |  | 0.005533638 |
| c0551 | Trigger factor | tig | 0.00268299 |
| c0559 | Hypothetical protein ybaW | ybaW | 0.003242404 |
| c0568 | Nitrogen Regulatory protein P-II 2 | glnK | 0.025037799 |
| c0577 | Maltose O-acetyltransferase | ylaD | 0.020352714 |
| c0581 | Acriflavine resistance protein A precursor | acrA | 0.016079251 |
| c0586 | Primosomal replication protein N | priC | 0.002440233 |
| c0588 | Adenine phosphoribosyltransferase | apt | 0.173739815 |
| c0602 | Hypothetical protein ybaP | ybaP | 0.032972011 |
| c0613 | Hypothetical protein ybbN | ybbN | 0.066901358 |
| c0618 | Hypothetical protein ybbB | ybbB | 0.085626519 |
| c0643 | Hypothetical protein ybcI | ybcI | 0.067549663 |
| c0649 | Tail fiber assembly protein homolog from lambdoid prophage Qin | ydfM | 0.006403051 |
| c0680 | Isochorismate synthase entC | entC | 0.19303254 |
| c0684 | Hypothetical protein ybdB | ybdB | 0 |
| c0701 | 2-(5''-triphosphoribosyl)-3'-dephosphocoenzyme-A synthase | citG | 0.102149716 |
| c0716 | Putative amidase | ybeM | 0.000564127 |
| c0789 | Hypothetical protein ybgI | ybgI | 0.014493517 |
| c0793 | Endonuclease VIII | nei | 0.153757751 |
| c0835 | UDP-glucose 4-epimerase | galE | 0.086853306 |
| c0841 | Molybdenum transport system permease protein modB | modB | 0.013019965 |
| c0858 | Dethiobiotin synthetase | bioD | 0.026380279 |
| c0872 | Hypothetical protein ybhO | ybhO | 0.016488037 |
| c0875 | Hypothetical protein ybhR | ybhR | 0.025033719 |
| c0885 | Hypothetical oxidoreductase ybiC | ybiC | 0.193452435 |
| c0889 | Hypothetical protein ybiX | ybiX | 0.051455827 |
| c0895 | Glutamine transport system permease protein glnP | glnP | 0.014444514 |
| c0896 | Glutamine-binding periplasmic protein precursor | glnH | 0.095088559 |
| c0900 | Outer membrane protein X precursor | ompX | 0.030356082 |
| c0913 | Putative L-asparaginase precursor | ybiK | 0.073656803 |
| c0929 | Protein ybjI |  | 0.041577703 |
| c0948 | Terminase, ATPase subunit |  | 0.194559808 |
| c0969 | Hypothetical protein yfdK | yfdK | 0.112384853 |
| c0982 | Glutaredoxin 1 | grxA | 0.007257736 |
| c0984 | Oxygen-insensitive NADPH nitroreductase | mdaA | 0.091984197 |
| c0997 | Arginine transport ATP-binding protein artP | artP | 0.073309692 |
| c1017 | Cold shock-like protein cspD | cspD | 0 |
| c1031 | Anaerobic dimethyl sulfoxide reductase chain A precursor | dmsA | 0.031963934 |
| c1068 | Hypothetical protein ycbK | ycbK | 0.004175003 |
| c1069 | Hypothetical protein ycbL | ycbL | 0.197179791 |
| c1077 | Putative aliphatic sulfonates transport permease protein ssuC | ycbM | 0.15804799 |
| c1098 | Hypothetical protein yccF | yccF | 0.00346328 |
| c1114 | Hydrogenase-1 large chain | hyaB | 0.109732802 |
| c1116 | Hydrogenase 1 maturation protease | hyaD | 0.004718806 |
| c1134 | Chaperone protein torD | torD | 0.002146005 |
| c1139 | Hypothetical protein yccJ | yccJ | 0 |
| c1175 | Putative aminotransferase |  | 0.03873023 |
| c1214 | Cea protein |  | 0.002406391 |
| c1223 |  |  | 0.091908108 |
| c1276 | Hypothetical protein |  | 0.055158073 |
| c1284 | Hypothetical protein yeeT |  | 0.123804052 |
| c1285 | Hypothetical protein |  | 0.192478491 |
| c1345 | Flagellar hook protein flgE | flgE | 0.017018994 |
| c1352 | Flagellar hook-associated protein 3 | flgL | 0.138332285 |
| c1376 | HIT-like protein ycfF | ycfF | 0 |
| c1423 | Hypothetical protein ydfU |  | 0.001794272 |
| c1428 | Hypothetical protein |  | 0.008573097 |
| c1440 | Hypothetical protein ydfO | ydfO | 0.136585372 |
| c1478 | Hypothetical protein |  | 0.022247782 |
| c1489 | Hypothetical protein |  | 0.144972642 |
| c1506 | Unknown protein encoded by prophage |  | 0.00550963 |
| c1513 | Putative Nudix hydrolase ymfB |  | 0.074474659 |
| c1528 | Hypothetical protein |  | 0.164826461 |
| c1540 | Lambda Regulatory protein CIII |  | 0.020254253 |
| c1545 | Hypothetical protein |  | 0.013284082 |
| c1599 | SitB protein | sitB | 0.015825755 |
| c1629 | Hypothetical protein ycgN | ycgN | 0.006941995 |
| c1639 | Alanine racemase, catabolic | dadX | 0.004743164 |
| c1703 | Thymidine kinase | tdk | 0.037281509 |
| c1708 | Oligopeptide transport system permease protein oppB | oppB | 0.130147151 |
| c1715 | Putative potassium channel protein | kch | 0.193327944 |
| c1760 | Putative transcriptional repressor |  | 0.146505435 |
| c1833 | Hypothetical protein |  | 0.067171832 |
| c1853 | Tellurite resistance protein tehA | tehA | 0.051925827 |
| c1878 | L-asparagine permease | ansP | 0.108919743 |
| c1887 | Hypothetical protein |  | 0.182493281 |
| c1899 | Respiratory nitrate reductase 2 beta chain | narY | 0.053238446 |
| c1911 | Alcohol dehydrogenase, propanol-preferring | adhP | 0.030711798 |
| c1914 | Bdm protein |  | 0.092201013 |
| c1991 | Hypothetical protein ydgE | ydgE | 0.003323342 |
| c2003 | Fumarate hydratase class II | fumC | 0.015843349 |
| c2010 | Uid operon repressor | uidR | 0.002616473 |
| c2058 | Hypothetical protein ydhQ | ydhQ | 0.140235597 |
| c2107 | Vitamin B12 transport system permease protein btuC | btuC | 0.199507974 |
| c2125 | Hypothetical protein yniB | yniB | 0.000631592 |
| c2166 | DNA topoisomerase III | topB | 0.125716602 |
| c2170 | Protease IV | sppA | 0.176376218 |
| c2247 | Serine/threonine protein phosphatase 1 | pphA | 0.022006979 |
| c2281 | Hypothetical isochorismatase family protein yecD | yecD | 0.007155546 |
| c2314 | L-arabinose-binding periplasmic protein precursor | araF | 0.034273255 |
| c2365 | Flagellar biosynthetic protein fliP precursor | fliP | 0.001011896 |
| c2370 | DsrB protein | dsrB | 0 |
| c2389 | Hypothetical protein yedY | yedY | 0.167779398 |
| c2417 | Hypothetical protein yeeI | yeeI | 0.032399914 |
| c2421 | Putative ABC transporter protein |  | 0.038176245 |
| c2472 | Transposase |  | 0 |
| c2560 | UDP-glucose 4-epimerase |  | 0.054081226 |
| c2561 | Hypothetical protein |  | 0.017374849 |
| c2571 | Lipopolysaccharide biosynthesis protein wzxC | wzxC | 0.114259655 |
| c2583 | Putative colanic acid biosynthesis glycosyl transferase wcaC | wcaC | 0.094962398 |
| c2588 | Hypothetical protein |  | 0.049702564 |
| c2602 | Hypothetical protein yegO | yegO | 0.185138438 |
| c2603 | Hypothetical transport protein yegB | yegB | 0.06523874 |
| c2606 | Hypothetical protein yegP | yegP | 0.054420416 |
| c2627 | Hypothetical sugar kinase yegV | yegV | 0.07322557 |
| c2641 | Mrp protein | mrp | 0.096991042 |
| c2653 | Hypothetical lipoprotein yehR precursor | yehR | 0.090074906 |
| c2654 | Hypothetical protein yehS | yehS | 0.004649764 |
| c2658 | Hypothetical ABC transporter permease protein yehW | yehW | 0.001752102 |
| c2665 | Penicillin-binding protein 7 precursor | pbpG | 0.005049225 |
| c2678 | Hypothetical protein yeiS | yeiS | 0.00287464 |
| c2687 | Hypothetical protein yeiB | yeiB | 0.182593459 |
| c2694 | Endonuclease IV | nfo | 0.04448137 |
| c2711 | Hypothetical protein yeiU | yeiU | 0.191727439 |
| c2717 | Hypothetical ABC transporter ATP-binding protein yejF | yejF | 0.116664344 |
| c2728 | Hypothetical protein |  | 0.003900547 |
| c2738 | Heme exporter protein A | ccmA | 0.003334154 |
| c2766 | Short-chain fatty acids transporter | atoE | 0.106245623 |
| c2790 | Hypothetical transcriptional regulator yfaX | yfaX | 0.076056768 |
| c2796 | Putative glycosyl transferase yfbF |  | 0.197238104 |
| c2809 | Menaquinone-specific isochorismate synthase | menF | 0.184334504 |
| c2844 | Hypothetical GST-like protein yfcF | yfcF | 0.156875243 |
| c2852 |  |  | 0.113779634 |
| c2872 | Hypothetical protein yfcM | yfcM | 0.092695811 |
| c2956 | Sulfate transport ATP-binding protein cysA | cysA | 0.022045281 |
| c2958 | Sulfate transport system permease protein cysT | cysU | 0.177225799 |
| c2975 | Ethanolamine ammonia-lyase heavy chain | eutB | 0.023716443 |
| c2978 | Ethanolamine utilization protein eutG | eutG | 0.17234991 |
| c3010 | Hypothetical protein |  | 0.000706337 |
| c3012 | Protein yfgD |  | 0.150741636 |
| c3032 | Hypothetical protein yfgJ | yfgJ | 0.134481718 |
| c3046 | Hypothetical protein |  | 0.05403651 |
| c3066 | Hypothetical protein yphB | yphB | 0.032923268 |
| c3075 | Flavohemoprotein | hmpA | 0.177847157 |
| c3206 | Hypothetical protein |  | 0.037820183 |
| c3252 | Regulatory protein recX | oraA | 0.118818621 |
| c3254 | Protein ygaD | ygaD | 0.013480406 |
| c3258 | PTS system, glucitol/sorbitol-specific IIA component | srlB | 0.001709516 |
| c3280 | Formate hydrogenlyase subunit 6 | hycF | 0.165747828 |
| c3293 | Hypothetical protein ygbA | ygbA | 0.134991319 |
| c3297 | Hypothetical oxidoreductase ygbJ | ygbJ | 0.125756616 |
| c3299 | Hypothetical aldolase class II protein ygbL | ygbL | 0.103014722 |
| c3312 | Protein ygbO | ygbO | 0.055680654 |
| c3358 | Hypothetical protein yqcC | yqcC | 0.177829293 |
| c3402 | Hypothetical protein |  | 0.1382606 |
| c3410 | Hypothetical protein |  | 0.195975846 |
| c3414 | Exodeoxyribonuclease V beta chain | recB | 0.00120132 |
| c3421 | Hypothetical protein |  | 0.005841037 |
| c3435 | Diaminopimelate decarboxylase | lysA | 0.136758265 |
| c3473 | Thiol:disulfide interchange protein dsbC precursor | dsbC | 0.083678482 |
| c3501 | Hypothetical protein yggA | yggA | 0.040689154 |
| c3507 | Hypothetical protein |  | 0.003276077 |
| c3509 | Putative ATP-binding protein of ABC transport system |  | 0.073849791 |
| c3512 | Hypothetical protein yggD | yggD | 0.001658467 |
| c3530 | Protein sprT | sprT | 0.044396943 |
| c3531 | Endonuclease I precursor | endA | 0.18843712 |
| c3546 | Hypothetical protein yggL | yggL | 0.01549435 |
| c3556 | Prophage P4 integrase |  | 0.112856905 |
| c3561 | Unknown protein encoded by ISEc8 |  | 0.048985033 |
| c3572 | Hypothetical protein |  | 0.004876857 |
| c3581 | Hypothetical protein |  | 0.149640301 |
| c3660 | Unknown protein encoded by ISEc8 |  | 0.017937004 |
| c3689 | 3-deoxy-manno-octulosonate cytidylyltransferase |  | 0.16913642 |
| c3708 |  |  | 0.027064691 |
| c3710 | Glc operon transcriptional activator | glcC | 0.153317118 |
| c3741 | Biopolymer transport exbB protein | exbB | 0.157075524 |
| c3743 | Hypothetical protein yghB | yghB | 0.044625607 |
| c3747 | Hypothetical protein yqhG precursor | yqhG | 0.124232055 |
| c3749 | Conserved hypothetical protein |  | 0.197140879 |
| c3772 | Putative iron compound permease protein of ABC transporter family |  | 0.153162511 |
| c3803 | Putative Transposase |  | 0.15276134 |
| c3804 | Hypothetical protein ydcM | ydcM | 0.145082798 |
| c3840 | Hypothetical protein ygjM |  | 0.187619667 |
| c3846 | Hypothetical protein ygjT | ygjT | 0.14240707 |
| c3858 | Hypothetical protein yqjK | yqjK | 0.003765062 |
| c3877 | Threonine dehydratase operon activator protein | tdcR | 0.141393236 |
| c3901 | Hypothetical protein yraN | yraN | 0.188654657 |
| c3903 | Hypothetical protein yraO | yraO | 0.191535785 |
| c3904 | Hypothetical protein yraP precursor | yraP | 0.023049685 |
| c3905 | Hypothetical protein yraQ | yraQ | 0.054445345 |
| c3911 | Putative protease yhbU precursor | yhbU | 0.088998534 |
| c3913 | Hypothetical protein yhbW | yhbW | 0.031878634 |
| c3916 | Cold-shock DEAD-box protein A | deaD | 0.106801507 |
| c3922 | tRNA pseudouridine synthase B | truB | 0.168716167 |
| c3941 | Hypothetical transport protein yhbE | yhbE | 0.130271907 |
| c3946 | Sugar fermentation stimulation protein B | nlp | 0.071562632 |
| c3951 | Hypothetical protein |  | 0.01199245 |
| c3967 | Hypothetical protein yrbL | yrbL | 0.080802504 |
| c3975 | Hypothetical protein yhcH | yhcH | 0.056444246 |
| c3983 | Hypothetical protein |  | 0.197878843 |
| c3987 | Hypothetical protein yhcB | yhcB | 0 |
| c3994 | Hypothetical protein yhcO | yhcO | 0.004094215 |
| c3997 | Hypothetical protein yhcR | yhcR | 0.00895911 |
| c4010 | Hypothetical protein |  | 0.036157728 |
| c4021 | Hypothetical protein |  | 0.146981906 |
| c4022 | Hypothetical protein yhdT | yhdT | 0.060989661 |
| c4041 | Hypothetical protein yrdB | yrdB | 0.00400761 |
| c4051 | Large-conductance mechanosensitive channel | mscL | 0.183668292 |
| c4052 | Putative regulator | yhdL | 0.027919429 |
| c4111 | Elongation factor Tu | tufA | 0.023898159 |
| c4135 | Para-aminobenzoate synthase glutamine amidotransferase component II | pabA | 0.098079754 |
| c4138 | Peptidyl-prolyl cis-trans isomerase A precursor | ppiA | 0.197553511 |
| c4143 | Potential nitrite transporter | nirC | 0.187010129 |
| c4153 | Hypothetical protein yhfZ | yhfZ | 0.036240407 |
| c4159 | 3-dehydroquinate synthase | aroB | 0.014328575 |
| c4180 | Osmolarity sensor protein envZ | envZ | 0.113535667 |
| c4181 | Transcriptional Regulatory protein ompR | ompR | 0.000596436 |
| c4182 | Hypothetical protein |  | 0.167217154 |
| c4190 | Hypothetical protein yhgH | yhgH | 0.075596145 |
| c4197 | RNA 3'-terminal phosphate cyclase |  | 0.173305866 |
| c4226 | Hypothetical protein |  | 0.134408423 |
| c4230 | Hypothetical protein |  | 0.188265144 |
| c4244 | High-affinity branched-chain amino acid transport ATP-binding protein livF | livF | 0.185300635 |
| c4259 | Hypothetical protein yhhL | yhhL | 0.016240663 |
| c4265 | DcrB protein precursor |  | 0.034096609 |
| c4292 | Universal stress protein B | yhiO | 0.126794379 |
| c4300 | Hypothetical protein |  | 0.011046608 |
| c4303 | Putative conserved protein |  | 0.160122029 |
| c4310 | Hypothetical protein |  | 0.172751549 |
| c4349 | Hypothetical protein yhjT | yhjT | 0.167776739 |
| c4353 | Hypothetical protein |  | 0.061877534 |
| c4365 | Hypothetical protein yhjX | yhjX | 0.186158911 |
| c4368 | Hypothetical acetyltransferase yiaC | yiaC | 0.146353336 |
| c4371 | Hypothetical protein |  | 0.028085043 |
| c4390 | BAX protein | bax | 0.077141483 |
| c4405 | Putative hexulose-6-phosphate isomerase | sgbU | 0.036360146 |
| c4413 | Hypothetical GST-like protein yibF | yibF | 0.006052843 |
| c4420 | Hypothetical protein |  | 0.09673394 |
| c4421 | Hypothetical protein yibL | yibL | 0 |
| c4448 | Lipid A-core, surface polymer ligase | waaL | 0.004801637 |
| c4449 | Putative beta1,3-glucosyltransferase | waaV | 0.15238148 |
| c4452 | Lipopolysaccharide 1,2-glucosyltransferase | rfaJ | 0.047527161 |
| c4476 | tRNA (Guanosine-2'-O-)-methyltransferase | spoU | 0.001379541 |
| c4477 | ATP-dependent DNA helicase recG | recG | 0.064679119 |
| c4481 | Conserved hypothetical protein |  | 0.183046427 |
| c4542 | Hypothetical protein |  | 0.155890797 |
| c4544 | Hypothetical protein |  | 0.126192435 |
| c4548 | Hypothetical protein |  | 0.04102463 |
| c4553 | Hypothetical protein |  | 0.13305348 |
| c4554 | Hypothetical protein |  | 0.080135592 |
| c4556 | Conserved hypothetical protein |  | 0.167745378 |
| c4560 | Hypothetical protein |  | 0.107728906 |
| c4599 | Hypothetical protein yidG | yidG | 0.091141007 |
| c4622 | DNA replication and repair protein recF | recF | 0.166895222 |
| c4647 | Hypothetical protein |  | 0.121689751 |
| c4670 | Protein mioC | mioC | 0.059856325 |
| c4686 | Protein yifE | yifE | 0.048917098 |
| c4697 | Peptidyl-prolyl cis-trans isomerase C | ppiC | 0.049214097 |
| c4703 | Hypothetical protein |  | 0.100488995 |
| c4726 | CyaY protein | cyaY | 0.060556112 |
| c4752 | Hypothetical protein |  | 0.070449305 |
| c4775 | Hypothetical protein |  | 0.188349706 |
| c4780 | Hypothetical protein |  | 0.024561988 |
| c4786 | Sec-independent protein translocase protein tatB |  | 0.000632363 |
| c4800 | Molybdopterin-guanine dinucleotide biosynthesis protein B | mobB | 0.004387015 |
| c4811 | Hypothetical protein |  | 0.162781396 |
| c4813 | Hypothetical protein |  | 0.086109275 |
| c4814 | Hypothetical protein |  | 0.020371898 |
| c4816 | Oxygen-independent coproporphyrinogen III oxidase | hemN | 0.06211606 |
| c4818 | Nitrogen regulation protein NR(II) | glnL | 0.121694613 |
| c4858 | Hypothetical protein |  | 0 |
| c4863 | Sensor protein cpxA | cpxA | 0.074475358 |
| c4886 | Cell division protein ftsN | ftsN | 0.128710908 |
| c4934 | Hypothetical protein |  | 0 |
| c4948 | Thiamin biosynthesis, probable sulfur donor | thiS | 0.022342388 |
| c4957 | DNA-binding protein HU-alpha | hupA | 0.135267561 |
| c4983 | PTS system, mannose-specific IIC component |  | 0.187224803 |
| c4985 | Putative sorbose PTS component |  | 0.128606673 |
| c4991 | Glucose-6-phosphate isomerase | pgi | 0.125802448 |
| c4999 | Hypothetical protein |  | 0.006367687 |
| c5005 | Maltose/maltodextrin transport ATP-binding protein malK | malK | 0.09093179 |
| c5012 | Hypothetical protein |  | 0.00502996 |
| c5014 | LexA repressor | lexA | 0.026276218 |
| c5046 | Hypothetical protein yjbQ | yjbQ | 0.018036751 |
| c5054 | Redox-sensitive transcriptional activator soxR | soxR | 0.026423905 |
| c5055 | Hypothetical protein |  | 0.002236605 |
| c5075 | Hypothetical protein |  | 0.188750285 |
| c5097 | Conserved hypothetical protein |  | 0 |
| c5112 | PhnB protein | phnB | 0.1958609 |
| c5126 |  |  | 0.023735195 |
| c5143 | Hypothetical protein |  | 0.063037622 |
| c5171 | Hypothetical protein |  | 0.054659578 |
| c5173 | Hypothetical protein |  | 0.13003472 |
| c5223 | FxsA protein |  | 0.066587737 |
| c5228 | Hypothetical protein yjeI precursor | yjeI | 0.011670998 |
| c5240 | Fumarate reductase 15 kDa hydrophobic protein | frdC | 0.005581229 |
| c5258 | HflK protein | hflK | 0.036705033 |
| c5285 | Probable hexulose-6-phosphate synthase | sgaH | 0.198923239 |
| c5287 | Putative hexulose-6-phosphate isomerase | sgaU | 0.112038483 |
| c5314 | Protein ytfJ precursor |  | 0.035100076 |
| c5315 | Hypothetical protein ytfK | ytfK | 0.004614885 |
| c5324 | Hypothetical protein |  | 0.006070837 |
| c5331 | UDP-N-acetylmuramate:L-alanyl-gamma-D-glutamyl- meso-diaminopimelate ligase | yjfG | 0.116862189 |
| c5352 | Protein yjgK | yjgK | 0.173356122 |
| c5354 | Hypothetical protein yjgD | yjgD | 0.041531268 |
| c5376 | Hypothetical protein |  | 0.135489074 |
| c5386 | Hypothetical protein |  | 0.076127409 |
| c5427 | Hypothetical protein yjiA | yjiA | 0.023047034 |
| c5431 | Hypothetical protein |  | 0.14121856 |
| c5442 | Protein yjjB | yjjB | 0.094573077 |
| c5443 | Hypothetical protein yjjP | yjjP | 0.180798754 |
| c5454 | Hypothetical protein yjjG | yjjG | 0.000572998 |
| c5472 | Protein smp precursor | smp | 0.190159538 |
| c5476 | Hypothetical protein |  | 0.017707162 |
| c5478 | ABC transporter ATP-binding protein yjjK | yjjK | 0.19620491 |
| c5481 | Hypothetical protein yjjX | yjjX | 0.086189223 |
| c5498 | IlvBN operon leader peptide | ivbL | 0.000940915 |
| c5500 | IlvGMEDA operon leader peptide | ilvL | 0.08893933 |
| c5502 | PyrBI operon leader peptide | pyrL | 0.02932669 |
| c5537 | tRNA-Arg |  | 0 |
| c5549 | tRNA-Asn | asnU | 0.022197122 |
| c5553 | tRNA-Ala | alaX | 0.046756666 |
| c5554 | tRNA-Ala | alaW | 0.020443629 |
| c5557 | tRNA-Val | valY | 0 |
| c5584 | tRNA-Ile | ileT | 0.095710379 |
