# Supplementary material for: Cyclic AMP Regulates Bacterial Persistence through Repression of the Oxidative Stress Response and SOS-Dependent DNA Repair in Uropathogenic Escherichia coli
Source: mBio. 2018 Jan 9;9(1):e02144-17. doi: 10.1128/mBio.02144-17 (PMC5760743; doi:10.1128/mBio.02144-17)
Supplement: TABLE S3 [file mbo001183668st3.docx]

**Table S3**

| Locus | Protein | Gene |
| --- | --- | --- |
|  |  |  |
| c0023 | Hypothetical protein |  |
| c0050 | FixA protein | *fixA* |
| c0070 | RNA polymerase associated protein | *hepA* |
| c0178 | DnaK suppressor protein | *dksA* |
| c0345 | Putative member of ShlA/HecA/FhaA exoprotein family |  |
| c0363 | Putative RTX family exoprotein A gene |  |
| c0393 | Haemoglobin protease |  |
| c0415 | Putative adhesin | *eaeH* |
| c0624 | 2-hydroxy-3-oxopropionate reductase | *ybbQ* |
| c0690 | Hypothetical protein ybdN |  |
| c0712 | Anaerobic C4-dicarboxylate transporter dcuC | *dcuC* |
| c0918 | Hypothetical protein yliE | *yliE* |
| c0924 | Penicillin-binding protein 6 precursor | *dacC* |
| c1163 | Hypothetical protein ycdS precursor | *ycdS* |
| c1267 | Hypothetical protein |  |
| c1541 | Putative single-stranded DNA binding protein of prophage |  |
| c1610 | Conserved hypothetical protein |  |
| c1726 | Tryptophan synthase beta chain | *trpB* |
| c1730 | Anthranilate synthase component I | *trpE* |
| c1745 | Aconitate hydratase 1 | *acnA* |
| c1780 | Putative sucrose phosphorylase | *ycjM* |
| c1802 | Hypothetical transcriptional regulator ycjZ |  |
| c1810 | Hypothetical protein |  |
| c1815 | Hypothetical protein ydaM | *ydaM* |
| c1821 | Unknown protein 2D_000B3L from 2D-page | *ynaF* |
| c1840 | ATP-dependent helicase hrpA | *hrpA* |
| c1842 | Aldehyde dehydrogenase A | *aldA* |
| c1880 | Putative conserved protein |  |
| c1901 | Nitrite extrusion protein 2 | *narU* |
| c1922 | Glutamate decarboxylase beta | *gadB* |
| c1930 | Hypothetical protein ydeP | *ydeP* |
| c1934 | Outer membrane usher protein fimD precursor |  |
| c1935 | Chaperone protein fimC precursor |  |
| c1962 | Hypothetical protein ydeE | *ydeF* |
| c1963 | Hypothetical protein ydeH | *ydeH* |
| c1987 | Hypothetical transport protein ynfM | *ynfM* |
| c2003 | Fumarate hydratase class II | *fumC* |
| c2009 | Beta-glucuronidase | *uidA* |
| c2082 | Hypothetical protein ydiJ | *ydiJ* |
| c2086 | Hypothetical transport protein ydiN | *ydiN* |
| c2089 | Hypothetical protein ydiF | *ydiF* |
| c2098 | Phosphoenolpyruvate synthase | *ppsA* |
| c2136 | PTS system, cellobiose-specific IIC component | *celB* |
| c2190 | Hypothetical protein yeaI | *yeaI* |
| c2256 | Protease II | *ptrB* |
| c2460 | Putative polyketide synthase |  |
| c2470 | Putative peptide/polyketide synthase |  |
| c2485 | Hypothetical protein ybdN |  |
| c2518 | TonB dependent receptor |  |
| c2555 | UDP-glucose 6-dehydrogenase | *ugd* |
| c2701 | Hypothetical sugar kinase yeiC | *yeiC* |
| c2775 | Hypothetical protein yfaL precursor | *yfaL* |
| c2813 | Hypothetical protein yfbK | *yfbK* |
| c2833 | Hypothetical protein yfbS | *yfbS* |
| c2899 | D-serine deaminase activator | *dsdC* |
| c2907 | Hypothetical protein yfdE | *yfdE* |
| c2939 | Xanthosine permease | *xapB* |
| c2995 | AegA protein | *yffG* |
| c3031 | SinH homolog |  |
| c3043 | Hypothetical lipoprotein yfhM precursor | *yfhM* |
| c3075 | Flavohemoprotein | *hmpA* |
| c3114 | ClpB protein | *clpB* |
| c3235 | Hypothetical protein ygaZ | *ygaZ* |
| c3332 | Hypothetical oxidoreductase ygcW | *ygcW* |
| c3415 | Protease III precursor | *ptr* |
| c3456 | Hypothetical protein ygfK | *ygfK* |
| c3465 | Putative purine permease ygfU | *ygfU* |
| c3607 | Hypothetical protein |  |
| c3652 | Hypothetical protein yfjI |  |
| c3712 | Putative saframycin Mx1 synthetase B |  |
| c3725 | Glutathionylspermidine synthase | *gsp* |
| c3792 | Hypothetical outer membrane usher protein yqiG precursor | *yqiG* |
| c3810 | Hypothetical transcriptional regulator ygiP | *ygiP* |
| c3876 | Tdc operon transcriptional activator | *tdcA* |
| c4289 | Hypothetical protein yhiM | *yhiM* |
| c4495 | Hexuronate transporter |  |
| c4631 | Tryptophanase | *tnaA* |
| c4832 | Hypothetical protein yihX | *yihX* |
| c4849 | Putative glycoporin |  |
| c4920 | Starvation sensing protein rspA |  |
| c4976 | 5-methyltetrahydrofolate--homocysteine methyltransferase | *metH* |
| c5202 | Regulatory protein | *pgtC* |
| c5244 | Hypothetical transporter yjeM | *yjeM* |
| c5341 | Mg(2+) transport ATPase, P-type 1 | *mgtA* |
