# Supplementary figures and images for: Cyclic AMP Regulates Bacterial Persistence through Repression of the Oxidative Stress Response and SOS-Dependent DNA Repair in Uropathogenic Escherichia coli
Source: mBio. 2018 Jan 9;9(1):e02144-17. doi: 10.1128/mBio.02144-17 (PMC5760743; doi:10.1128/mBio.02144-17)

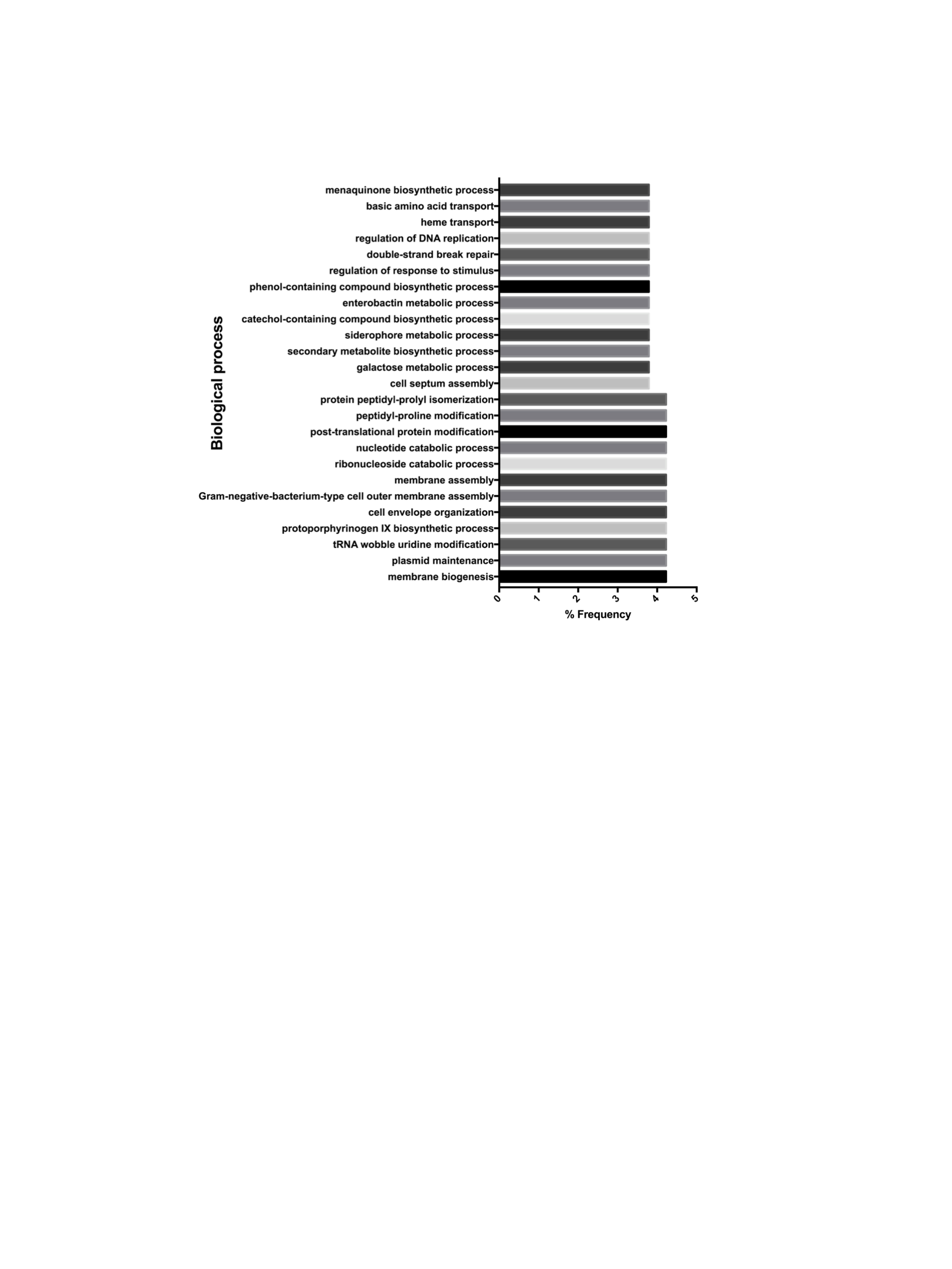

Supplement: FIG S1 [file mbo001183668sf1.tif]

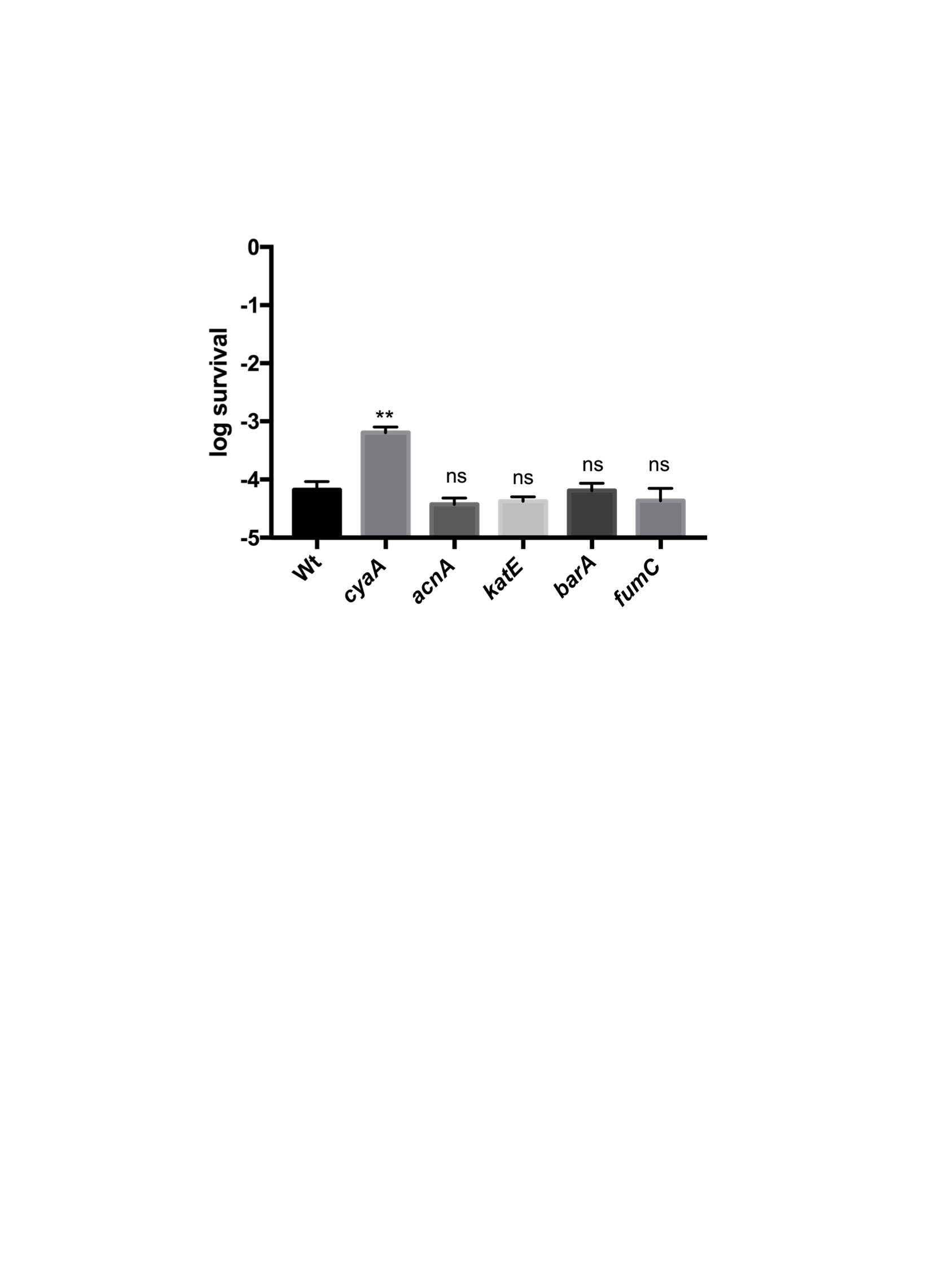

Supplement: FIG S2 [file mbo001183668sf2.tif]

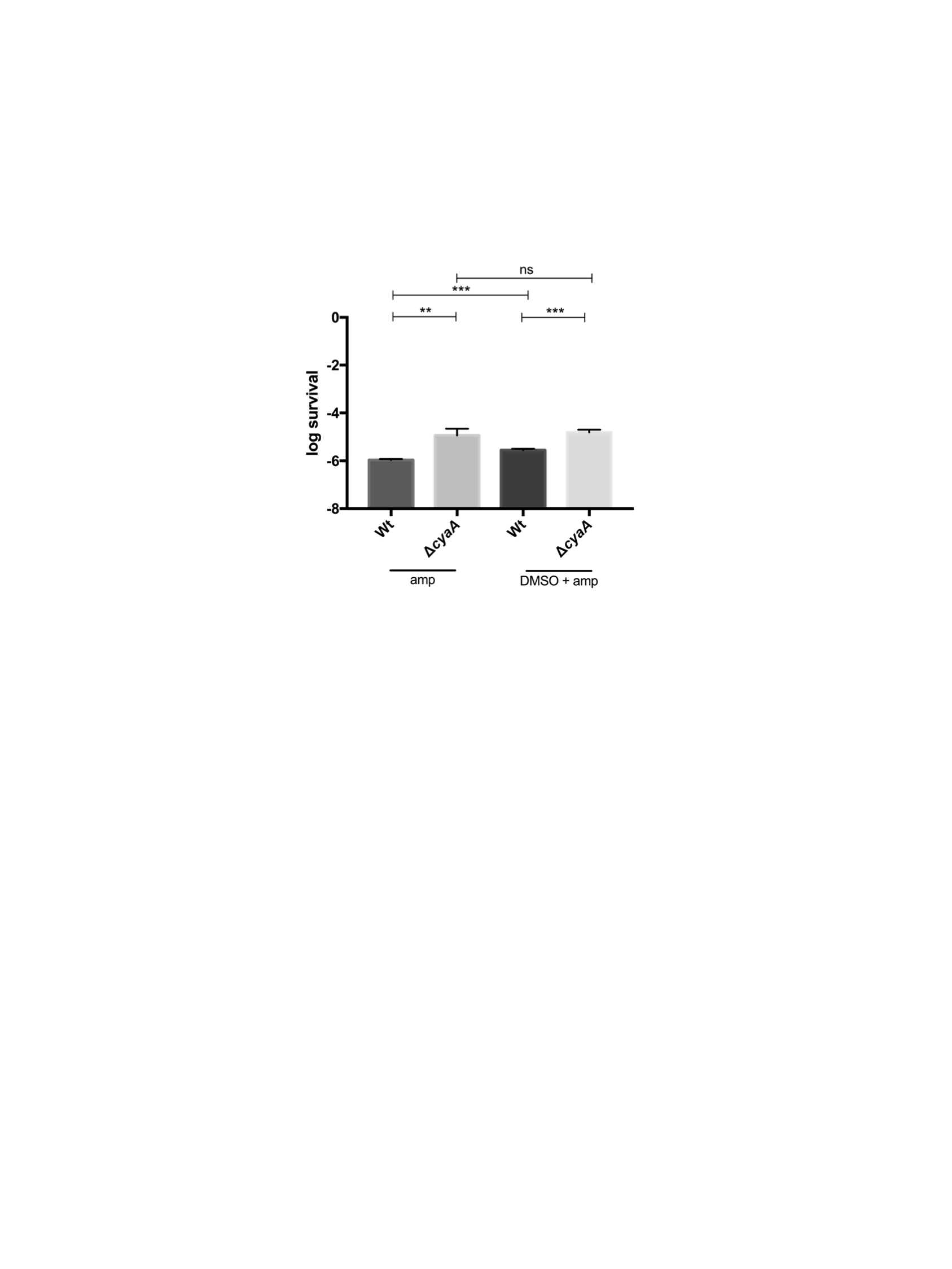

Supplement: FIG S3 [file mbo001183668sf3.tif]

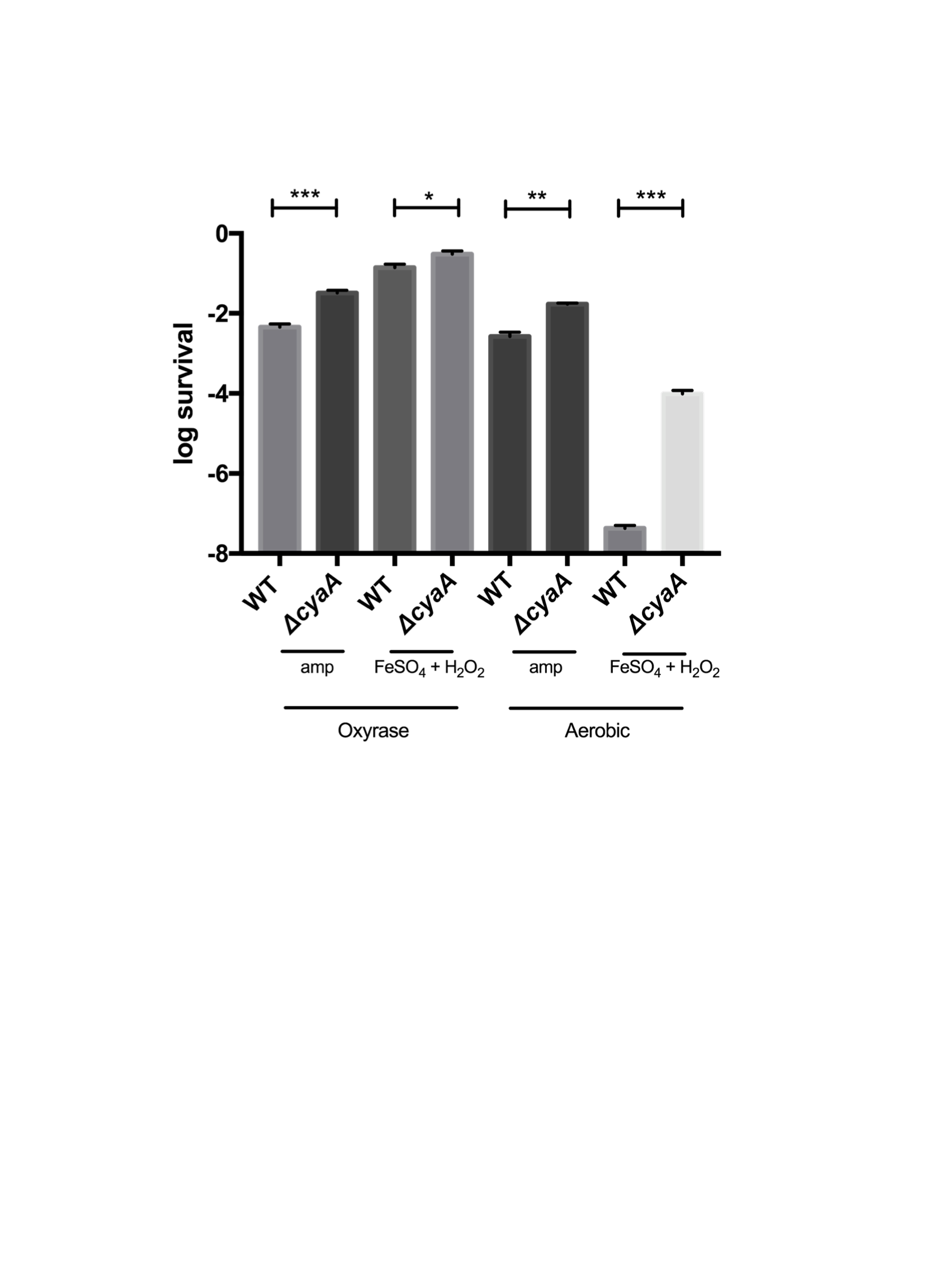

Supplement: FIG S4 [file mbo001183668sf4.tif]
