# Supplementary material for: Cyclic AMP Regulates Bacterial Persistence through Repression of the Oxidative Stress Response and SOS-Dependent DNA Repair in Uropathogenic Escherichia coli
Source: mBio. 2018 Jan 9;9(1):e02144-17. doi: 10.1128/mBio.02144-17 (PMC5760743; doi:10.1128/mBio.02144-17)
Supplement: TABLE S4 [file mbo001183668st4.docx]

**Table S4**

| Strain/plasmid | Relevant genotype or characteristic(s) | Source |
| --- | --- | --- |
|  |  |  |
| CFT073 | *E. coli* wild type, clinical isolate | Laboratory stock |
| cyaA | CFT073 Δ*cyaA*::FRT | Laboratory stock |
| cyaA/Tn library | EZ-Tn5™ <R6Kγori /KAN-2> transposon library in *E. coli* *cyaA*::FRT background | This study |
| katE::kan | CFT073 Δ*katE*::*aph*, Kan^R^ | This study |
| acnA::kan | CFT073 Δ*acnA*::*aph*, Kan^R^ | This study |
| barA::kan | CFT073 Δ*barA*::*aph*, Kan^R^ | This study |
| fumC::kan | CFT073 Δ*fumC*::*aph*, Kan^R^ | This study |
| eaeH::kan | CFT073 Δ*eaeH*::*aph*, Kan^R^ | This study |
| umuC::kan | CFT073 Δ*umuC*::*aph*, Kan^R^ | This study |
| cyaA katE::kan | CFT073 Δ*cyaA*::FRT Δ*katE*::*aph*, Kan^R^ | This study |
| cyaA acnA::kan | CFT073 Δ*cyaA*::FRT Δ*acnA*::*aph*, Kan^R^ | This study |
| cyaA barA::kan | CFT073 Δ*cyaA*::FRT Δ*barA*::*aph*, Kan^R^ | This study |
| cyaA fumC::kan | CFT073 Δ*cyaA*::FRT Δ*fumC*::*aph*, Kan^R^ | This study |
| cyaA eaeH::kan | CFT073 Δ*cyaA*:: Δ*eaeH*::*aph*, Kan^R^ | This study |
| cyaA umuC::kan | CFT073 Δ*cyaA*::FRT Δ*umuC*::*aph*, Kan^R^ | This study |
| pKM208 | IPTG-inducible lambda red recombineering system, Amp^R^ | This study |
| pDL1093 | Temperature-sensitive mTn*10* delivery vector, CmR, SpR | Laboratory stock |
| pKD4 | Contains kanamycin selection marker used to construct mutants | Laboratory stock |
|  |  | Laboratory stock |
|  |  |  |
|  |  |  |
|  |  |  |
|  |  |  |
|  |  |  |
|  |  |  |
