# Supplementary material for: Cyclic AMP Regulates Bacterial Persistence through Repression of the Oxidative Stress Response and SOS-Dependent DNA Repair in Uropathogenic Escherichia coli
Source: mBio. 2018 Jan 9;9(1):e02144-17. doi: 10.1128/mBio.02144-17 (PMC5760743; doi:10.1128/mBio.02144-17)
Supplement: TABLE S6 [file mbo001183668st6.docx]

**Table S6**

| Name | Sequence (5’- 3’ orientation) | Purpose |
| --- | --- | --- |
|  |  |  |
| olj363 | GTGTGGGCACTCGACATATGACAAG | Anneals with Tn10 end in pDL1093 |
| C-tail | GTGACTGGAGTTCAGACGTGTGCTCTTCCGATCTGGGGGGGGGGGGGGGG | C-tail complementary |
| olj385 | AATGATACGGCGACCACCGAGATCTACACTCTTTGGGGGCCAAAATCATTAGGGGATTCATCAG | Nested PCR (2nd PCR) |
| olj386 | ACACTCTTTGGGGGCCAAAATCATTAGGGGATTCATCAG | Sequencing Primer |
| BC45G | CAAGCAGAAGACGGCATACGAGATTGTTGACTGTGACTGGAGTTCAGACGTGTGCTCTTCCGATCTGGGGGGGGGGGGGGGG | agtcaaca (barcode) |
| BC46G | CAAGCAGAAGACGGCATACGAGATACGGAACTGTGACTGGAGTTCAGACGTGTGCTCTTCCGATCTGGGGGGGGGGGGGGGG | agttccgt (barcode) |
| BC47G | CAAGCAGAAGACGGCATACGAGATTCTGACATGTGACTGGAGTTCAGACGTGTGCTCTTCCGATCTGGGGGGGGGGGGGGGG | atgtcaga (barcode) |
| BC49G | CAAGCAGAAGACGGCATACGAGATGTGCGGACGTGACTGGAGTTCAGACGTGTGCTCTTCCGATCTGGGGGGGGGGGGGGGG | gtccgcac (barcode) |
| BC50G | CAAGCAGAAGACGGCATACGAGATCGTTTCACGTGACTGGAGTTCAGACGTGTGCTCTTCCGATCTGGGGGGGGGGGGGGGG | gtgaaacg (barcode) |
| BC51G | CAAGCAGAAGACGGCATACGAGATAAGGCCACGTGACTGGAGTTCAGACGTGTGCTCTTCCGATCTGGGGGGGGGGGGGGGG | gtggcctt (barcode) |
| BC52G | CAAGCAGAAGACGGCATACGAGATACCGAAACGTGACTGGAGTTCAGACGTGTGCTCTTCCGATCTGGGGGGGGGGGGGGGG | gtttcggt (barcode) |
| BC53G | CAAGCAGAAGACGGCATACGAGATTACGTACGGTGACTGGAGTTCAGACGTGTGCTCTTCCGATCTGGGGGGGGGGGGGGGG | cgtacgta (barcode) |
| BC54G | CAAGCAGAAGACGGCATACGAGATATCCACTCGTGACTGGAGTTCAGACGTGTGCTCTTCCGATCTGGGGGGGGGGGGGGGG | gagtggat (barcode) |
| BC55G | CAAGCAGAAGACGGCATACGAGATATATCAGTGTGACTGGAGTTCAGACGTGTGCTCTTCCGATCTGGGGGGGGGGGGGGGG | actgatat (barcode) |
| BC56G | CAAGCAGAAGACGGCATACGAGATAAAGGAATGTGACTGGAGTTCAGACGTGTGCTCTTCCGATCTGGGGGGGGGGGGGGGG | attccttt (barcode) |
| F_Kan_katE | ATGTCGCAACATAACGAAAAGAACCCACATCAGCACCAGTGTGTAGGCTGGAGCTGCTTC | Forward primer to construct the mutant with a kanamycin selection marker |
| R_Kan_katE | TTACGCCGGGATTTTGTCAATCTTAGGAATGCGTGACCACCATATGAATATCCTCCTTAG | Reverse primer to construct the mutant with a kanamycin selection marker |
| F_check_katE | ACCGTTAATCCTGATTTGTTACGTT | Check the site-specific recombination of the kanamycin marker |
| F_Kan_acnA | ATGTCGTCAACCCTACGAGAAGCCAGTAAAGACACGTTGCGTGTAGGCTGGAGCTGCTTC | Forward primer to construct the mutant with a kanamycin selection marker |
| R_Kan_acnA | TTACTTCAACATATTACGAATGACATAATGCAAAATGCCGCATATGAATATCCTCCTTAG | Reverse primer to construct the mutant with a kanamycin selection marker |
| F_check_acnA | GTCACCATTATGTCAGCATAGTGAC | Check the site-specific recombination of the kanamycin marker |
| F_Kan_barA | ATGACCAACTACAGCCTGCGCGCACGCATGATGATTCTGAGTGTAGGCTGGAGCTGCTTC | Forward primer to construct the mutant with a kanamycin selection marker |
| R_Kan_barA | ATTACCCGAGAATTTTGCTGGCTTCGCGCGCTACATTATCCATATGAATATCCTCCTTAG | Reverse primer to construct the mutant with a kanamycin selection marker |
| F_check_barA | AAACCCCATCTGAAGTTGCTGTGTT | Check the site-specific recombination of the kanamycin marker |
| F_Kan_fumC | ATGAATACAGTACGCAGCGAAAAAGATTCGATGGGGGCGAGTGTAGGCTGGAGCTGCTTC | Forward primer to construct the mutant with a kanamycin selection marker |
| R_Kan_fumC | TTAACCCCCGGCTTTCATACTGCCGACCATCTGTTCTGGCCATATGAATATCCTCCTTAG | Reverse primer to construct the mutant with a kanamycin selection marker |
| F_check_fumC | TTATGCCTCCGGTTCTCTTGGCCCA | Check the site-specific recombination of the kanamycin marker |
| F_Kan_eaeH | ATGTCACGTTATAAAACAGACAATAAACAGCCACGATTTCGTGTAGGCTGGAGCTGCTTC | Forward primer to construct the mutant with a kanamycin selection marker |
| R_Kan_eaeH | TTATTTCTCCTCAGCGCCTTCAGTATCTGCAGGAACGGCGCATATGAATATCCTCCTTAG | Reverse primer to construct the mutant with a kanamycin selection marker |
| F_check_eaeH | GGATTTGTCAGGGGTTATGAACAAA | Check the site-specific recombination of the kanamycin marker |
| F_Kan_umuC | ATGTTTGCCCTCTGTGATGTAAACGCGTTTTATGCCAGCTGTGTAGGCTGGAGCTGCTTC | Forward primer to construct the mutant with a kanamycin selection marker |
| R_Kan_umuC | TTATTTGACCCGCAGTAAATCAGAACTTCGCGTTGTATAACATATGAATATCCTCCTTAG | Reverse primer to construct the mutant with a kanamycin selection marker |
| F_check_umuC | TTGATAGCCTGAATCAGTATTGATC | Check the site-specific recombination of the kanamycin marker |
